# Strong convergence theorem for split monotone variational inclusion with constraints of variational inequalities and fixed point problems

**DOI:** 10.1186/s13660-018-1905-6

**Published:** 2018-11-15

**Authors:** Jin-Lin Guan, Lu-Chuan Ceng, Bing Hu

**Affiliations:** 1 Department of Mathematics, Shanghai Normal University, Shanghai, China; 2 LAMPS and Department of Mathematics and Statistics, York University, Toronto, Canada

**Keywords:** 47H05, 47H09, 47H10, 47J22, Strong convergence, Iterative scheme, Split monotone variational inclusion, Variational inequality, Fixed point, *k*-strict pseudo-contractions, Hilbert spaces

## Abstract

In this paper, inspired by Jitsupa et al. (J. Comput. Appl. Math. 318:293–306, 2017), we propose a general iterative scheme for finding a solution of a split monotone variational inclusion with the constraints of a variational inequality and a fixed point problem of a finite family of strict pseudo-contractions in real Hilbert spaces. Under very mild conditions, we prove a strong convergence theorem for this iterative scheme. Our result improves and extends the corresponding ones announced by some others in the earlier and recent literature.

## Introduction

It is known that variational inequality, as a greatly important tool, has already been studied for a wide class of unilateral, obstacle, and equilibrium problems arising in several branches of pure and applied sciences in a unified and general framework. Many numerical methods have been developed for solving variational inequalities and some related optimization problems; see [2–5] and the references therein.

The split monotone variational inclusion problem, which is the core of the modeling of many inverse problems arising in phase retrieval and other real-world problems, has been widely studied in sensor networks, intensity-modulated radiation therapy treatment planning, data compression, and computerized tomography in recent years; see, e.g., [6–10] and the references therein.

Split monotone variational inclusion problem (in short, SMVIP) was firstly introduced by Moudafi [11] as follows: find $x^{\ast}\in H_{1}$ such that

1.1 $$ \textstyle\begin{cases} 0\in f_{1}x^{\ast}+B_{1}x^{\ast}, \\ y^{\ast}=Ax^{\ast}\in H_{2}: 0\in f_{2}y^{\ast}+B_{2}y^{\ast}, \end{cases} $$

where $f_{1}: H_{1}\rightarrow H_{1}$ and $f_{2}: H_{2}\rightarrow H_{2}$ are two given single-valued mappings, $A: H_{1}\rightarrow H_{2}$ is a bounded linear operator, $B_{1}: H_{1}\rightarrow 2^{H_{1}}$ and $B_{2}: H_{2}\rightarrow2^{H_{2}}$ are multi-valued maximal monotone mappings.

If $f_{1}=f_{2}\equiv0$, then problem (1.1) reduces to the following split variational inclusion problem (in short, SVIP): find $x^{\ast }\in H_{1}$ such that

1.2 $$ \textstyle\begin{cases} 0\in B_{1}x^{\ast}, \\ y^{\ast}=Ax^{\ast}\in H_{2}: 0\in B_{2}y^{\ast}. \end{cases} $$

Also, if $f_{1}\equiv0$, then problem (1.1) reduces to the following split monotone variational inclusion problem (in short, SMVIP): find $x^{\ast}\in H_{1}$ such that

1.3 $$ \textstyle\begin{cases} 0\in B_{1}x^{\ast}, \\ y^{\ast}=Ax^{\ast}\in H_{2}: 0\in fy^{\ast}+B_{2}y^{\ast}. \end{cases} $$

We denote the solution sets of variational inclusions $0\in B_{1}x^{\ast}$ and $0\in fy^{\ast}+B_{2}y^{\ast}$ by $\operatorname{SOLVIP}(B_{1})$ and $\operatorname{SOLVIP}(f+B_{2})$, respectively. Thus, the solution set of problem (1.3) can be denoted by $\varGamma= \{x^{\ast}\in H_{1}: x^{\ast}\in \operatorname{SOLVIP}(B_{1}), Ax^{\ast}\in \operatorname{SOLVIP}(f+B_{2})\}$.

In 2012, Byrne et al. [12] studied the following iterative scheme for SVIP (1.2): for given $x_{0}\in H_{1}$ and $\lambda> 0$,

1.4 $$ x_{n+1}=J^{B_{1}}_{\lambda} \bigl[x_{n}+\epsilon A^{\ast} \bigl(J^{B_{2}}_{\lambda}-I \bigr)Ax_{n} \bigr] . $$

Recently, Kazmi and Rivi [13] introduced a new iterative scheme for SVIP (1.2) and the fixed point problem of a nonexpansive mapping:

1.5 $$ \textstyle\begin{cases} u_{n}=J^{B_{1}}_{\lambda}[x_{n}+\epsilon A^{\ast}(J^{B_{2}}_{\lambda }-I)Ax_{n}], \\ x_{n+1}=\alpha_{n}f(x_{n})+(1-\alpha _{n})Tu_{n}, \end{cases} $$

where *A* is a bounded linear operator, $A^{\ast}$ is the adjoint of *A*, *f* is a contraction on $H_{1}$, *T* is a nonexpansive mapping of $H_{1}$. They obtained a strong convergence theorem under some mild restrictions on the parameters.

Very recently, Jitsupa et al. [1] modified algorithm (1.5) for SVIP (1.2) and the fixed point problem of a finite family of strict pseudo-contractions:

1.6 $$ \textstyle\begin{cases} u_{n}=J^{B_{1}}_{\lambda}[x_{n}+\gamma A^{\ast}(J^{B_{2}}_{\lambda }-I)Ax_{n}], \\ y_{n}=\beta_{n}u_{n}+(1-\beta_{n})\sum^{N} _{i=1}\eta _{i}^{(n)}T_{i}u_{n}, \\ x_{n+1}=\alpha_{n}\tau f(x_{n})+(I-\alpha_{n}D)y_{n},\quad n\geq 1, \end{cases} $$

where *A* is a bounded linear operator, $A^{\ast}$ is the adjoint of *A*, $\{T_{i}\}^{N}_{i=1}$ is a finite family of $k_{i}$-strictly pseudo-contractions, *f* is a contraction, *D* is a strong positive linear bounded operator. They proved, under certain appropriate assumptions on the sequences $\{\alpha_{n}\}$, $\{\beta_{n}\}$, and $\{\eta_{i}^{(n)}\}_{i=1}^{N}$, that $\{x_{n}\}$ defined by (1.6) converges strongly to a common solution of SVIP (1.2) and a fixed point of a finite family of $k_{i}$-strictly pseudo-contractions, which solves some variational inequality problem.

### Remark 1.1

1. We notice that Jitsupa et al. [1] did not define the domains and the ranges of $B_{1}$ and $B_{2}$ in the iteration process (1.6) and Theorem 3.1 of [1]. Certainly, it is easy to misunderstand that $B_{1}$ is defined on $H_{1}$ into $2^{H_{1}}$ and $B_{2}$ is defined on $H_{2}$ into $2^{H_{2}}$. In that case, $\{u_{n}\}$ defined in (1.6) lies in $H_{1}$. However, the domain of $T_{i}$ is *C* but not $H_{1}$, which makes the iteration process (1.6) not well-defined. Thus, it is necessary to give the definite domains and ranges of $B_{1}$ and $B_{2}$.
2. Can the iterative scheme (1.6) be modified for solving more problems?

In this paper, we introduce a new general iterative scheme as follows:

1.7 $$ \textstyle\begin{cases} u_{n}=J^{B_{1}}_{\lambda_{1}}[x_{n}+\gamma A^{\ast }(J^{B_{2}}_{\lambda_{2}}(I-\lambda_{2}f)-I)Ax_{n}], \\ v_{n}=P_{C}(u_{n}-\xi Du_{n}), \\ y_{n}=\beta_{n}v_{n}+(1-\beta_{n})\sum^{N} _{i=1}\eta _{i}^{(n)}T_{i}v_{n}, \\ x_{n+1}=P_{C}[\alpha_{n}\tau F(x_{n})+\gamma_{n}x_{n}+((1-\gamma _{n})I-\alpha_{n}\mu V)y_{n}], \quad n\geq 1, \end{cases} $$

where $B_{1}: C\rightarrow2^{H_{1}}$, $B_{2}: H_{2}\rightarrow 2^{H_{2}}$ are two multi-valued maximal monotone operators, $f: H_{2}\rightarrow H_{2}$ is a *ρ*-inverse strongly monotone operator, $A: H_{1}\rightarrow H_{2}$ is a bounded linear operator, and $A^{\ast}$ is the adjoint of *A*, $D: C\rightarrow H_{1}$ is a *δ*-inverse strongly monotone operator, $\{T_{i}\}^{N}_{i=1}: C\rightarrow C$ is a finite family of $k_{i}$-strictly pseudo-contractions, $P_{C}$ is the metric projection of $H_{1}$ onto the closed convex set *C*, *F* is *L*-Lipschitzian on $H_{1}$, and *V* is a *η*-strongly monotone and *K*-Lipschitzian operator. Under some suitable assumptions on the sequences $\{\alpha_{n}\}$, $\{\beta _{n}\}$, and $\{\eta_{i}^{(n)}\}_{i=1}^{N}$, we prove that the sequence $\{x_{n}\}$ defined by (1.7) converges strongly to a common solution of SMVIP (1.3) with the constraints of a variational inequality and a fixed point problem of a finite family of strict pseudo-contractions, which solves the following variational inequality:

$$ \langle\mu Vq-\tau Fq, q-p\rangle\leq 0, \quad \forall p\in\mathcal{F} , $$

where $\mathcal{F}$ denotes the set of common solutions of SMVIP (1.3), a variational inequality, and a fixed point problem of a finite family of strict pseudo-contractions. Finally, we also provide a numerical example to support our strong convergence result.

## Preliminaries

Throughout this paper, let $H_{1}$ and $H_{2}$ be two real Hilbert spaces with the inner product $\langle\cdot, \cdot\rangle$ and the norm $\|\cdot\|$. Let *C* be a nonempty closed convex subset of $H_{1}$.

Recall that $S : H_{1}\rightarrow H_{1}$ is said to be a nonexpansive mapping if $\|Sx-Sy\|\leq\|x-y\|$, $\forall x,y\in H_{1}$. It is also called firmly nonexpansive if $\langle Sx-Sy, x-y\rangle\geq\|Sx-Sy\| ^{2}$, $\forall x,y\in H_{1}$. We can easily see that *S* is firmly nonexpansive if and only if *S* can be written as $S=\frac {1}{2}(I+T)$, where $T : H_{1}\rightarrow H_{1}$ is nonexpansive.

Moreover, $S : H_{1}\rightarrow H_{1}$ is called

(i) contractive if there exists a constant $\alpha\in(0, 1)$ such that
  2.1 $$ \|Sx-Sy\|\leq\alpha\|x-y\|, \quad \forall x,y\in H_{1} ; $$
(ii) *L*-Lipschitzian if there exists a positive constant *L* such that
  2.2 $$ \|Sx-Sy\|\leq L\|x-y\|,\quad \forall x,y\in H_{1} ; $$
(iii) *η*-strongly monotone if there exists a positive constant *η* such that
  2.3 $$ \langle Sx-Sy, x-y\rangle\geq\eta\|x-y\| ^{2}, \quad \forall x,y\in H_{1} ; $$
(iv) *β*-inverse strongly monotone (in short, *β*-*ism*) if there exists a positive constant *β* such that
  2.4 $$ \langle Sx-Sy, x-y\rangle\geq\beta\|Sx-Sy\| ^{2}, \quad \forall x,y\in H_{1} ; $$
(v) averaged if it can be expressed as the average of the identity mapping and a nonexpansive mapping, i.e.,
  2.5 $$ S:=(1-\alpha)I+\alpha T , $$

where $\alpha\in(0,1)$, *I* is the identity operator on $H_{1}$ and $T: H_{1}\rightarrow H_{1}$ is nonexpansive.

It is easily seen that averaged mappings are nonexpansive. In the meantime, firmly nonexpansive mappings are averaged.

In addition, a mapping $S : H_{1}\rightarrow H_{1}$ is called *k*-strict pseudo-contractive if there exists a constant $k\in[0,1)$ such that

2.6 $$ \Vert Sx-Sy \Vert ^{2}\leq \Vert x-y \Vert ^{2}+k \bigl\Vert (I-S)x-(I-S)y \bigr\Vert ^{2}, \quad \forall x,y\in H_{1} . $$

A linear operator *D* is said to be a strongly positive bounded linear operator on $H_{1}$ if there exists a positive constant *τ̅* such that

$$ \langle Dx, x\rangle\geq\overline{\tau}\| x\|^{2}, \quad \forall x\in H_{1} . $$

From the definition above, we obtain easily that a strongly positive bounded linear operator *D* is *τ̅*-strongly monotone and $\|D\|$-Lipschitzian.

A multi-valued mapping $M: D(M)\subseteq H_{1}\rightarrow2^{H_{1}}$ is called monotone if, for all $x, y\in D(M)$, $u\in Mx$ and $v\in My$ such that

$$ \langle x-y, u-v\rangle\geq0 . $$

A monotone mapping *M* is maximal if the $\operatorname{Graph}(M)$ is not properly contained in the graph of any other monotone mapping. It is well known that a monotone mapping *M* is maximal if and only if for $x\in D(M)$, $u\in H_{1}$, $\langle x-y, u-v\rangle\geq0$ for each $(y, v)\in \operatorname{Graph}(M)$ implies that $u\in Mx$.

Let $M: D(M)\subseteq H_{1}\rightarrow2^{H_{1}}$ be a multi-valued maximal monotone mapping. Then the resolvent operator $J^{M}_{\lambda }: H_{1}\rightarrow D(M)$ is defined by

$$ J^{M}_{\lambda}x:= (I+\lambda M)^{-1}(x), \quad \forall x\in H_{1} , $$

for $\forall\lambda>0$, where *I* stands for the identity operator on $H_{1}$. We observe that $J^{M}_{\lambda}$ is single-valued, nonexpansive, and firmly nonexpansive.

Let $D: C\rightarrow H_{1}$ be a nonlinear mapping. Then the variational inequality problem (VIP) is to find $u\in C$ such that

2.7 $$ \langle Du, v-u\rangle\geq0,\quad \forall v\in C . $$

We denote the solution set of VIP (2.7) by $\operatorname{VI}(C, D)$. Many different approaches have been studied for solving this problem; see, e.g., [14–17].

For each point $x\in H_{1}$, there exists a unique nearest point in *C* denoted by $P_{C}x$ such that

2.8 $$ \|x-P_{C}x\|\leq\|x-y\|, \quad \forall y\in C . $$

$P_{C}$ is called the metric projection of $H_{1}$ onto *C*.

It is known that $P_{C}$ is nonexpansive and satisfies the following inequalities:

2.9 $$\begin{aligned}& \|P_{C}x-P_{C}y\|^{2}\leq\langle x-y, P_{C}x-P_{C}y\rangle, \quad \forall x, y\in H_{1} , \end{aligned}$$

2.10 $$\begin{aligned}& \langle x-P_{C}x, y-P_{C}x\rangle\leq 0, \quad \forall x \in H_{1}, y\in C. \end{aligned}$$

We note that each nonexpansive mapping $S: H_{1}\rightarrow H_{1}$ satisfies the following inequality (see Theorem 3 in [18] and Theorem 1 in [19]):

2.11 $$ \bigl\langle (x-Sx)-(y-Sy), Sy-Sx \bigr\rangle \leq \frac{1}{2} \bigl\Vert (Sx-x)-(Sy-y) \bigr\Vert ^{2}, \quad \forall x, y\in H_{1} , $$

particularly, for $\forall x\in H_{1}$, $y\in F(S)$,

2.12 $$ \langle x-Sx, y-Sx\rangle\leq\frac{1}{2}\| Sx-x\|^{2} . $$

### Proposition 2.1

([11])

(i) *If* $T=(1-\alpha)S+\alpha V$, *where* $S: H_{1}\rightarrow H_{1}$ *is averaged*, $V: H_{1}\rightarrow H_{1}$ *is nonexpansive*, *and* $\alpha\in [0, 1]$, *then* *T* *is averaged*.
(ii) *The composite of finitely many averaged mappings is averaged*.
(iii) *If the mappings* $\{T_{i}\}^{N}_{i=1}$ *are averaged and have a nonempty common fixed point*, *then*
  $$ \bigcap^{N} _{i=1}F(T_{i})=F(T_{1} \circ T_{2}\circ\cdots\circ T_{N}) . $$
(iv) *If* *T* *is* *ν*-*ism*, *then for* $\gamma>0$, *γT* *is* $\frac {\nu}{\gamma}$-*ism*.
(v) *T* *is averaged if and only if its complement* $I-T$ *is* *ν*-*ism for some* $\nu>\frac{1}{2}$.

### Proposition 2.2

([11])

*Let* $\lambda>0$, *h* *be an* *α*-*ism operator*, *and* *B* *be a maximal monotone operator*. *If* $\lambda\in(0, 2\alpha)$, *then it is easily seen that the operator* $J^{B}_{\lambda}(I-\lambda h)$ *is averaged*.

### Proposition 2.3

([11])

*Let* $\lambda>0$ *and* $B_{1}$ *be a maximal monotone operator*. *Then*

$$ x^{\ast} \textit{ solves }(1.1) \quad \Leftrightarrow\quad x^{\ast}=J^{B_{1}}_{\lambda}(I-\lambda f_{1}) \bigl(x^{\ast} \bigr)\quad \textit{and}\quad Ax^{\ast}=J^{B_{2}}_{\lambda}(I- \lambda f_{2})Ax^{\ast} . $$

### Proposition 2.4

([20])

*Let* $D: C\rightarrow H_{1}$ *be an inverse strongly monotone operator*. *Then*

$$ u\in \operatorname{VI}(C, D)\quad \Leftrightarrow\quad u=P_{C}(u- \lambda Du), \quad \forall\lambda>0 . $$

### Proposition 2.5

([21])

*Let* *D* *be an inverse strongly*-*monotone mapping of* *C* *into* $H_{1}$. *Let* $N_{C}v$ *be the normal cone to* *C* *at* $v\in C$, *i*.*e*.,

$$ N_{C}v= \bigl\{ w\in H_{1}| \langle v-u, w\rangle \geq0, \forall u \in C \bigr\} , $$

*and define*

$$ Tv= \textstyle\begin{cases} Dv+N_{C}v,& v\in C, \\ \emptyset,& v\in H_{1}\setminus C. \end{cases} $$

*Then* *T* *is maximal monotone and* $0\in Tv$ *if and only if* $v\in \operatorname{VI}(C, D)$.

In order to prove our main results, we need the following lemmas.

### Lemma 2.1

([22])

*Let* $T: C\rightarrow C$ *be a* *k*-*strict pseudo*-*contraction*. *For* $\lambda\in[k, 1)$, *define* $S: C\rightarrow C$ *by* $Sx=\lambda x+(1-\lambda)Tx$ *for each* $x\in C$. *Then* *S* *is a nonexpansive mapping such that* $F(S)=F(T)$.

### Lemma 2.2

([23])

*If* $T: C\rightarrow C$ *is a* *k*-*strict pseudo*-*contraction*, *then the fixed point set* $F(T)$ *is closed convex so that the projection* $P_{F(T)}$ *is well*-*defined*.

### Lemma 2.3

([23])

*Let* *C* *be a nonempty closed convex subset of the Hilbert space* $H_{1}$. *Given an integer* $N\geq1$, *assume that* $\{T_{i}\}^{N}_{i=1}: C\rightarrow C$ *is a finite family of* $k_{i}$-*strict pseudo*-*contractions*. *Suppose that* $\{\eta_{i}\} ^{N}_{i=1}$ *is a positive sequence such that* $\sum^{N}_{i=1}\eta _{i}=1$. *Then* $\sum^{N}_{i=1}\eta_{i}T_{i}: C\rightarrow C$ *is a* *k*-*strict pseudo*-*contraction with* $k=\max\{k_{i}: 1\leq i\leq N\}$ *and* $F(\sum^{N}_{i=1}\eta_{i}T_{i})=\bigcap^{N}_{i=1}F(T_{i})$.

### Lemma 2.4

([24])

*Let* *E* *be an inner product space*. *Then*, *for any* $x, y, z\in E$ *and* $\alpha, \beta, \gamma\in[0, 1]$ *with* $\alpha+\beta+\gamma=1$, *we have*

$$ \|\alpha x+\beta y+\gamma z\|^{2}=\alpha\|x\| ^{2}+\beta\|y \|^{2}+\gamma\|z\|^{2}-\alpha\beta\|x-y\|^{2}-\alpha \gamma\|x-z\|^{2}-\beta\gamma\|y-z\|^{2} . $$

### Lemma 2.5

([25])

*Let* $\{\alpha_{n}\}$ *be a sequence of nonnegative numbers satisfying the property*

$$ \alpha_{n+1}\leq(1-\gamma_{n})\alpha _{n}+ \gamma_{n}\delta_{n}, \quad n\geq0 , $$

*where* $\{\gamma_{n}\}$ *is a sequence in* $(0, 1)$ *and* $\{\delta_{n}\}$ *is a real sequence in* $\mathbb{R}$ *such that*

(i) $\sum^{\infty}_{n=1}\gamma_{n}=\infty$;
(ii) $\limsup_{n\rightarrow\infty}\delta_{n}\leq0$ *or* $\sum^{\infty}_{n=1}|\gamma_{n}\delta_{n}|<\infty$.

*Then* $\lim_{n\rightarrow\infty}\alpha_{n}=0$.

### Lemma 2.6

([26])

*Assume that* *T* *is nonexpansive self*-*mapping of a closed convex subset* *C* *of a Hilbert space* $H_{1}$. *If* *T* *has a fixed point*, *then* $I-T$ *is demiclosed*, *i*.*e*., *whenever* $\{ x_{n}\}$ *weakly converges to some* *x* *and* $\{(I-T)x_{n}\}$ *converges strongly to* *y*, *it follows that* $(I-T)x=y$. *Here* *I* *is the identity mapping on* $H_{1}$.

### Lemma 2.7

([27])

*Let* *V* *be a* *K*-*Lipschitzian and* *η*-*strongly monotone operator on a nonempty closed convex subset* *C* *of a Hilbert space* $H_{1}$ *with* $0<\eta\leq K$ *and* $0< t<2\eta /K^{2}$. *Then the mapping* $S: C\rightarrow C$ *defined by* $S:=(I-tV)$ *is a contraction with coefficient* $\tau_{t}=1-t(\eta-\frac{tK^{2}}{2})$.

### Lemma 2.8

([28])

*Let* *C* *be a nonempty closed convex subset of a Hilbert space* $H_{1}$ *and* $P_{C}$ *be the metric projection of* $H_{1}$ *onto* *C*. *Let* $S : C\rightarrow C$ *be a nonexpansive mapping with* $F(S)\neq\emptyset$ *and* $F: C\rightarrow H_{1}$ *be an* *L*-*Lipschitzian mapping with constant* $L\geq0$. *Let* $V: C\rightarrow H_{1}$ *be an* *η*-*strongly monotone and* *K*-*Lipschitzian mapping*. *Suppose that* $0<\mu<2\eta/K^{2}$ *and* $0\leq\tau L<\tau_{0}$, *where* $\tau_{0}=1-\sqrt{1-\mu(2\eta-\mu K^{2})}$. *Then the net* $\{x_{t}\} _{t\in(0, 1)}$ *defined by* $x_{t}=P_{C}[t\tau Fx_{t}+(I-t\mu V)Sx_{t}]$ *converges strongly as* $t\rightarrow0$ *to a fixed point* *q* *of* *S* *which solves the variational inequality*

$$ \bigl\langle (\mu V-\tau F)q, q-p \bigr\rangle \leq 0, \quad \forall p\in F(S) . $$

## Main results

### Lemma 3.1

*Let* $H_{1}$ *and* $H_{2}$ *be two real Hilbert spaces and* *C* *be a nonempty closed convex subset of* $H_{1}$. *Let* $A: H_{1}\rightarrow H_{2}$ *be a bounded linear operator*, $A^{\ast }$ *be the adjoint of* *A*, *and* *r* *be the spectral radius of the operator* $A^{\ast}A$. *Let* $f: H_{2}\rightarrow H_{2}$ *be a* *ρ*-*inverse strongly monotone operator and* $B_{1}: C\rightarrow 2^{H_{1}}$, $B_{2}: H_{2}\rightarrow2^{H_{2}}$ *be two multi*-*valued maximal monotone operators*. *Let* $D: C\rightarrow H_{1}$ *be a* *δ*-*inverse strongly monotone operator*. *Assume that* $\{T_{i}\}^{N}_{i=1}: C\rightarrow C$ *is a finite family of* $k_{i}$-*strict pseudo*-*contraction mappings such that* $\mathcal{F}:=\bigcap^{N}_{i=1}F(T_{i})\cap\varGamma \cap \operatorname{VI}(C, D)\neq\emptyset$. *Let* $P_{C}$ *be the metric projection of* $H_{1}$ *onto* *C*, *and* $F: C\rightarrow H_{1}$ *be an* *L*-*Lipschitzian mapping with constant* $L\geq0$. *Suppose that* $V: C\rightarrow H_{1}$ *is an* *η*-*strongly monotone and* *K*-*Lipschitzian mapping with* $0<\eta\leq K$, $0<\mu<2\eta/K^{2}$ *and* $0\leq\tau L<\tau_{0}$, *where* $\tau_{0}=1-\sqrt{1-\mu(2\eta-\mu K^{2})}$. *For* $x_{1}\in C$, *let* $\{x_{n}\}$ *be a sequence of* *C* *generated by* (1.7). *Assume that the following conditions hold*:

(i) $\lambda_{1}>0$, $0<\lambda_{2}<2\rho$, $0<\gamma<\frac{1}{r}$, $0<\xi<2\delta$;
(ii) $0<\alpha_{n}<1$, $\sum^{\infty}_{i=1}\alpha_{n}=\infty$, $\lim_{n\rightarrow\infty}\alpha_{n}=0$;
(iii) $\max_{1\leq i\leq N}k_{i}\leq\beta_{n}\leq l<1$, $\lim_{n\rightarrow\infty}\beta_{n}=l$;
(iv) $\sum^{N}_{i=1}\eta^{(n)}_{i}=1$, $0<\gamma_{n}<1$, $\lim_{n\rightarrow\infty}\gamma_{n}=0$;
(v) $\sum^{\infty}_{n=1}(|\alpha_{n+1}-\alpha_{n}|+|\beta _{n+1}-\beta_{n}|+|\gamma_{n+1}-\gamma_{n}|+\sum^{N}_{i=1}|\eta ^{(n+1)}_{i}-\eta^{(n)}_{i}|)<\infty$.

*Then* $\lim_{n\rightarrow\infty}\|x_{n+1}-x_{n}\|=0$.

### Proof

Let $G_{n}:=\sum^{N}_{i=1}\eta^{(n)}_{i}T_{i}$. By Lemma 2.3, we obtain that, for each $n\geq1$, $G_{n}$ is a *k*-strict pseudo-contraction on *C* and $F(G_{n})=\bigcap^{N}_{i=1}F(T_{i})$, where $k=\max\{k_{i}: 1\leq i\leq N\}$. Let $U:=J^{B_{2}}_{\lambda _{2}}(I-\lambda_{2}f)$. Then the iterative scheme (1.7) can be rewritten as

3.1 $$ \textstyle\begin{cases} u_{n}=J^{B_{1}}_{\lambda_{1}}[x_{n}+\gamma A^{\ast}(U-I)Ax_{n}], \\ v_{n}=P_{C}(u_{n}-\xi Du_{n}), \\ y_{n}=\beta_{n}v_{n}+(1-\beta_{n})G_{n}v_{n}, \\ x_{n+1}=P_{C}[\alpha_{n}\tau Fx_{n}+\gamma_{n}x_{n}+((1-\gamma _{n})I-\alpha_{n}\mu V)y_{n}],\quad n\geq 1. \end{cases} $$

We divide the rest of the proof into two steps.

*Step* 1. We claim that the sequence $\{x_{n}\}$ is bounded.

Indeed, take $p\in\mathcal{F}$. Then $J^{B_{1}}_{\lambda_{1}}p=p$, $U(Ap)=Ap$, $G_{n}p=p$, $P_{C}(I-\xi D)p=p$, and it is easily seen that $Wp=p$, where $W:=I+\gamma A^{\ast}(U-I)A$. From the definition of firm nonexpansion and Proposition 2.2, we have that $J^{B_{1}}_{\lambda _{1}}$ and *U* are averaged. Likewise *W* is also averaged because it is $\frac{\nu}{r}$-*ism* for some $\nu>\frac{1}{2}$. Actually, by (v) of Proposition 2.1, we know that $I-U$ is *ν*-*ism* with $\nu >\frac{1}{2}$. Hence, we have

$$\begin{aligned} \bigl\langle A^{\ast}(I-U)Ax-A^{\ast}(I-U)Ay, x-y \bigr\rangle &= \bigl\langle (I-U)Ax-(I-U)Ay, Ax-Ay \bigr\rangle \\ &\geq\nu \bigl\Vert (I-U)Ax-(I-U)Ay \bigr\Vert ^{2} \\ &\geq\frac{\nu}{r} \bigl\Vert A^{\ast}(I-U)Ax-A^{\ast}(I-U)Ay \bigr\Vert ^{2}. \end{aligned}$$

Thus $\gamma A^{\ast}(I-U)A$ is $\frac{\nu}{\gamma r}$-*ism*. Due to the condition $0<\gamma<\frac{1}{r}$, the complement $I-\gamma A^{\ast}(I-U)A$ is averaged, and so is $M:=J^{B_{1}}_{\lambda_{1}}[I+\gamma A^{\ast}(U-I)A]$. Therefore, $J^{B_{1}}_{\lambda_{1}}$, *U*, *W*, and *M* are nonexpansive mappings.

From (3.1), we estimate

3.2 $$\begin{aligned} \Vert u_{n}-p \Vert ^{2}&= \bigl\Vert J^{B_{1}}_{\lambda_{1}} \bigl[x_{n}+\gamma A^{\ast }(U-I)Ax_{n} \bigr]-J^{B_{1}}_{\lambda_{1}}p \bigr\Vert ^{2} \\ &\leq \bigl\Vert x_{n}+\gamma A^{\ast}(U-I)Ax_{n}-p \bigr\Vert ^{2} \\ &= \Vert x_{n}-p \Vert ^{2}+\gamma^{2} \bigl\Vert A^{\ast}(U-I)Ax_{n} \bigr\Vert ^{2}+2\gamma \bigl\langle x_{n}-p, A^{\ast}(U-I)Ax_{n} \bigr\rangle . \end{aligned}$$

Thus, we get

3.3 $$ \|u_{n}-p\|^{2}\leq\|x_{n}-p\|^{2}+ \gamma ^{2} \bigl\langle (U-I)Ax_{n}, AA^{\ast}(U-I)Ax_{n} \bigr\rangle +2\gamma \bigl\langle x_{n}-p, A^{\ast}(U-I)Ax_{n} \bigr\rangle . $$

Next, setting $\varLambda_{1}:=\gamma^{2}\langle(U-I)Ax_{n}, AA^{\ast }(U-I)Ax_{n}\rangle$, we estimate

3.4 $$\begin{aligned} \varLambda_{1}&=\gamma^{2} \bigl\langle (U-I)Ax_{n}, AA^{\ast }(U-I)Ax_{n} \bigr\rangle \\ &\leq r\gamma^{2} \bigl\langle (U-I)Ax_{n}, (U-I)Ax_{n} \bigr\rangle \\ &=r\gamma^{2} \bigl\Vert (U-I)Ax_{n} \bigr\Vert ^{2}. \end{aligned}$$

Setting $\varLambda_{2}:=2\gamma\langle x_{n}-p, A^{\ast }(U-I)Ax_{n}\rangle$, we obtain from (2.12)

3.5 $$\begin{aligned} \varLambda_{2}&=2\gamma \bigl\langle x_{n}-p, A^{\ast}(U-I)Ax_{n} \bigr\rangle \\ &=2\gamma \bigl\langle A(x_{n}-p), (U-I)Ax_{n} \bigr\rangle \\ &=2\gamma \bigl\langle A(x_{n}-p)+(U-I)Ax_{n}-(U-I)Ax_{n}, (U-I)Ax_{n} \bigr\rangle \\ &=2\gamma \bigl( \bigl\langle UAx_{n}-Ap, (U-I)Ax_{n} \bigr\rangle - \bigl\Vert (U-I)Ax_{n} \bigr\Vert ^{2} \bigr) \\ &\leq2\gamma \biggl(\frac{1}{2} \bigl\Vert (U-I)Ax_{n} \bigr\Vert ^{2}- \bigl\Vert (U-I)Ax_{n} \bigr\Vert ^{2} \biggr) \\ &\leq-\gamma \bigl\Vert (U-I)Ax_{n} \bigr\Vert ^{2}. \end{aligned}$$

In view of (3.3)–(3.5), we have

3.6 $$ \|u_{n}-p\|^{2}\leq\|x_{n}-p\|^{2}+ \gamma (r\gamma-1) \bigl\Vert (U-I)Ax_{n} \bigr\Vert ^{2}. $$

From $0<\gamma<\frac{1}{r}$, we obtain

3.7 $$ \|u_{n}-p\|\leq\|x_{n}-p\|. $$

Since *D* is *δ*-inverse strongly monotone and $0<\xi<2\delta$, we estimate

$$\begin{aligned} \Vert v_{n}-p \Vert ^{2}&= \bigl\Vert P_{C}(I-\xi D)u_{n}-P_{C}(I-\xi D)p \bigr\Vert ^{2} \\ &\leq \bigl\Vert (I-\xi D)u_{n}-(I-\xi D)p \bigr\Vert ^{2} \\ &= \bigl\Vert (u_{n}-p)-\xi(Du_{n}-Dp) \bigr\Vert ^{2} \\ &= \Vert u_{n}-p \Vert ^{2}-2\xi\langle Du_{n}-Dp, u_{n}-p\rangle+\xi^{2} \Vert Du_{n}-Dp \Vert ^{2} \\ &\leq \Vert u_{n}-p \Vert ^{2}-2\xi\delta \Vert Du_{n}-Dp \Vert ^{2}+\xi^{2} \Vert Du_{n}-Dp \Vert ^{2} \\ &= \Vert u_{n}-p \Vert ^{2}+\xi(\xi-2\delta) \Vert Du_{n}-Dp \Vert ^{2} \\ &\leq \Vert u_{n}-p \Vert ^{2}, \end{aligned}$$

which implies

3.8 $$ \|v_{n}-p\|\leq\|u_{n}-p\|. $$

Define $S_{n}x:=\beta_{n}x+(1-\beta_{n})G_{n}x$, $\forall x\in C$. Using Lemma 2.1, we obtain that $S_{n} : C\rightarrow C$ is a nonexpansive mapping and $F(S_{n})=F(G_{n})$. It is clear that $S_{n}p=p$, and hence

3.9 $$ \|y_{n}-p\|=\|S_{n}v_{n}-p\|=\| S_{n}v_{n}-S_{n}p\|\leq\|v_{n}-p \|. $$

By (3.7)–(3.9), we have

3.10 $$ \|y_{n}-p\|\leq\|v_{n}-p\|\leq\|u_{n}-p\| \leq \|x_{n}-p\|. $$

It follows from (3.1) and Lemma 2.7 that

$$\begin{aligned} &\Vert x_{n+1}-p \Vert \\ &\quad = \bigl\Vert P_{C} \bigl[ \alpha_{n}\tau Fx_{n}+\gamma _{n}x_{n}+ \bigl((1-\gamma_{n})I-\alpha_{n}\mu V \bigr)y_{n} \bigr]-P_{C}p \bigr\Vert \\ &\quad \leq \bigl\Vert \alpha_{n}\tau Fx_{n}+ \gamma_{n}x_{n}+ \bigl((1-\gamma _{n})I- \alpha_{n}\mu V \bigr)y_{n}-p \bigr\Vert \\ &\quad = \bigl\Vert \alpha_{n}(\tau Fx_{n}-\mu Vp)+ \gamma_{n}(x_{n}-p)+ \bigl[(1-\gamma _{n})I- \alpha_{n}\mu V \bigr]y_{n}- \bigl[(1-\gamma_{n})I- \alpha_{n}\mu V \bigr]p \bigr\Vert \\ &\quad \leq \bigl\Vert \bigl[(1-\gamma_{n})I-\alpha_{n}\mu V \bigr]y_{n}- \bigl[(1-\gamma _{n})I-\alpha_{n}\mu V \bigr]p \bigr\Vert +\gamma_{n} \Vert x_{n}-p \Vert + \alpha_{n} \Vert \tau Fx_{n}-\mu Vp \Vert \\ &\quad \leq \biggl[1-\gamma_{n}-\alpha_{n}\mu \biggl(\eta- \frac{\alpha_{n}\mu K^{2}}{2(1-\gamma_{n})} \biggr) \biggr] \Vert y_{n}-p \Vert + \gamma_{n} \Vert x_{n}-p \Vert +\alpha _{n} \Vert \tau Fx_{n}-\mu Vp \Vert \\ &\quad \leq \biggl[1-\gamma_{n}-\alpha_{n}\mu \biggl(\eta- \frac{\alpha_{n}\mu K^{2}}{2(1-\gamma_{n})} \biggr) \biggr] \Vert x_{n}-p \Vert + \gamma_{n} \Vert x_{n}-p \Vert +\alpha _{n} \Vert \tau Fx_{n}-\mu Vp \Vert \\ &\quad = \biggl[1-\alpha_{n}\mu \biggl(\eta-\frac{\alpha_{n}\mu K^{2}}{2(1-\gamma _{n})} \biggr) \biggr] \Vert x_{n}-p \Vert +\alpha_{n} \Vert \tau Fx_{n}-\mu Vp \Vert \\ &\quad \leq \biggl[1-\alpha_{n}\mu \biggl(\eta-\frac{\alpha_{n}\mu K^{2}}{2(1-\gamma _{n})} \biggr) \biggr] \Vert x_{n}-p \Vert +\alpha_{n} \bigl[ \Vert \tau Fx_{n}-\tau Fp \Vert + \Vert \tau Fp-\mu Vp \Vert \bigr] \\ &\quad \leq \biggl[1-\alpha_{n}\mu \biggl(\eta-\frac{\alpha_{n}\mu K^{2}}{2(1-\gamma _{n})} \biggr) \biggr] \Vert x_{n}-p \Vert +\alpha_{n}\tau L \Vert x_{n}-p \Vert +\alpha_{n} \Vert \tau Fp-\mu Vp \Vert \\ &\quad = \biggl[1-\alpha_{n} \biggl(\mu\eta-\frac{\alpha_{n}\mu K^{2}}{2(1-\gamma _{n})}-\tau L \biggr) \biggr] \Vert x_{n}-p \Vert +\alpha_{n} \Vert \tau Fp- \mu Vp \Vert . \end{aligned}$$

By induction, we derive

$$ \|x_{n}-p\|\leq\max \bigl\{ \Vert x_{0}-p \Vert , M_{1} \bigr\} , $$

where $M_{1}=\sup_{n\geq1}\frac{\|\tau Fp-\mu Vp\|}{\mu\eta-\frac {\alpha_{n}\mu K^{2}}{2(1-\gamma_{n})}-\tau L}$. This shows that $\{ x_{n}\}$ is bounded, and so are $\{y_{n}\}$, $\{v_{n}\}$, and $\{u_{n}\}$.

*Step* 2. We claim $\|x_{n+1}-x_{n}\|\rightarrow0$ as $n\rightarrow\infty$.

Indeed, from (3.1), we have

3.11 $$\begin{aligned} & \Vert x_{n+1}-x_{n} \Vert \\ &\quad = \bigl\Vert P_{C} \bigl[\alpha_{n}\tau Fx_{n}+\gamma_{n}x_{n}+ \bigl((1-\gamma _{n})I-\alpha_{n}\mu V \bigr)y_{n} \bigr] \\ &\qquad {} -P_{C} \bigl[\alpha_{n-1}\tau Fx_{n-1}+ \gamma_{n-1}x_{n-1}+ \bigl((1-\gamma _{n-1})I- \alpha_{n-1}\mu V \bigr)y_{n-1} \bigr] \bigr\Vert \\ &\quad \leq \bigl\Vert \alpha_{n}\tau Fx_{n}+ \gamma_{n}x_{n}+ \bigl((1-\gamma _{n})I- \alpha_{n}\mu V \bigr)y_{n} \\ &\qquad {} -\alpha_{n-1}\tau Fx_{n-1}-\gamma_{n-1}x_{n-1}- \bigl((1-\gamma _{n-1})I-\alpha_{n-1}\mu V \bigr)y_{n-1} \bigr\Vert \\ &\quad \leq \bigl\Vert \bigl((1-\gamma_{n})I-\alpha_{n} \mu V \bigr)y_{n}- \bigl((1-\gamma _{n-1})I- \alpha_{n-1}\mu V \bigr)y_{n-1} \bigr\Vert + \alpha_{n}\tau \Vert Fx_{n}-Fx_{n-1} \Vert \\ & \qquad {} + \Vert \gamma_{n}x_{n}- \gamma_{n-1}x_{n-1} \Vert +|\alpha_{n}-\alpha _{n-1}| \Vert \tau Fx_{n-1} \Vert \\ &\quad \leq \bigl\Vert \bigl((1-\gamma_{n})I-\alpha_{n} \mu V \bigr)y_{n}- \bigl((1-\gamma _{n})I- \alpha_{n}\mu V \bigr)y_{n-1} \bigr\Vert + \bigl\Vert \bigl((1-\gamma_{n})I-\alpha_{n}\mu V \bigr)y_{n-1} \\ & \qquad {} - \bigl((1-\gamma_{n-1})I-\alpha_{n-1}\mu V \bigr)y_{n-1} \bigr\Vert +\alpha_{n}\tau L \Vert x_{n}-x_{n-1} \Vert + \Vert \gamma_{n}x_{n}- \gamma_{n}x_{n-1} \Vert \\ &\qquad {} + \Vert \gamma_{n}x_{n-1}-\gamma_{n-1}x_{n-1} \Vert +|\alpha_{n}-\alpha _{n-1}| \Vert \tau Fx_{n-1} \Vert \\ &\quad \leq \biggl[1-\gamma_{n}-\alpha_{n}\mu \biggl(\eta- \frac{\alpha_{n}\mu K^{2}}{2(1-\gamma_{n})} \biggr) \biggr] \Vert y_{n}-y_{n-1} \Vert \\ &\qquad {} +|\gamma_{n}-\gamma _{n-1}| \Vert y_{n-1} \Vert +|\alpha_{n}-\alpha_{n-1}| \Vert \mu Vy_{n-1} \Vert +\alpha_{n}\tau L \Vert x_{n}-x_{n-1} \Vert \\ &\qquad {} +\gamma_{n} \Vert x_{n}-x_{n-1} \Vert +|\gamma_{n}-\gamma_{n-1}| \Vert x_{n-1} \Vert +|\alpha_{n}-\alpha_{n-1}| \Vert \tau Fx_{n-1} \Vert \\ &\quad = \biggl[1-\gamma_{n}-\alpha_{n}\mu \biggl(\eta- \frac{\alpha_{n}\mu K^{2}}{2(1-\gamma_{n})} \biggr) \biggr] \Vert y_{n}-y_{n-1} \Vert +(\gamma_{n}+\alpha _{n}\tau L) \Vert x_{n}-x_{n-1} \Vert \\ &\qquad {} +|\alpha_{n}-\alpha_{n-1}| \bigl( \Vert \mu Vy_{n-1} \Vert + \Vert \tau Fx_{n-1} \Vert \bigr)+| \gamma_{n}-\gamma_{n-1}| \bigl( \Vert x_{n-1} \Vert + \Vert y_{n-1} \Vert \bigr) \\ &\quad \leq \biggl[1-\gamma_{n}-\alpha_{n}\mu \biggl(\eta- \frac{\alpha_{n}\mu K^{2}}{2(1-\gamma_{n})} \biggr) \biggr] \Vert y_{n}-y_{n-1} \Vert +(\gamma_{n}+\alpha _{n}\tau L) \Vert x_{n}-x_{n-1} \Vert \\ &\qquad {} +|\alpha_{n}-\alpha_{n-1}|M_{2}+| \gamma_{n}-\gamma _{n-1}|M_{3}, \end{aligned}$$

where $M_{2}=\sup_{n\geq1}\{\|\mu Vy_{n-1}\|+\|\tau Fx_{n-1}\|\}$, $M_{3}=\sup_{n\geq1}\{\|x_{n-1}\|+\|y_{n-1}\|\}$. Furthermore, since $y_{n}=S_{n}v_{n}$, we have

3.12 $$\begin{aligned} &\Vert y_{n}-y_{n-1} \Vert \\ &\quad = \Vert S_{n}v_{n}-S_{n-1}v_{n-1} \Vert \\ &\quad \leq \Vert S_{n}v_{n}-S_{n}v_{n-1} \Vert + \Vert S_{n}v_{n-1}-S_{n-1}v_{n-1} \Vert \\ &\quad \leq \Vert v_{n}-v_{n-1} \Vert + \bigl\Vert \beta_{n}v_{n-1}+(1-\beta _{n})G_{n}v_{n-1}- \bigl[\beta_{n-1}v_{n-1}+(1-\beta_{n-1})G_{n-1}v_{n-1} \bigr] \bigr\Vert \\ &\quad = \Vert v_{n}-v_{n-1} \Vert + \bigl\Vert ( \beta_{n}-\beta _{n-1}) (v_{n-1}-G_{n-1}v_{n-1})+(1- \beta _{n}) (G_{n}v_{n-1}-G_{n-1}v_{n-1}) \bigr\Vert \\ &\quad \leq \Vert v_{n}-v_{n-1} \Vert +|\beta_{n}- \beta_{n-1}| \Vert v_{n-1}-G_{n-1}v_{n-1} \Vert +(1-\beta_{n}) \Vert G_{n}v_{n-1}-G_{n-1}v_{n-1} \Vert \\ &\quad \leq \Vert v_{n}-v_{n-1} \Vert +|\beta_{n}- \beta_{n-1}|M_{4}+\sum^{N} _{i=1} \bigl\vert \eta^{(n)}_{i}- \eta^{(n-1)}_{i} \bigr\vert \Vert T_{i}v_{n-1} \Vert , \end{aligned}$$

where $M_{4}=\sup_{n\geq1}\|v_{n-1}-G_{n-1}v_{n-1}\|$.

By the nonexpansion of $P_{C}$ and $I-\xi D$, we get

3.13 $$\begin{aligned} \Vert v_{n}-v_{n-1} \Vert &= \bigl\Vert P_{C}(I-\xi D)u_{n}-P_{C}(I-\xi D)u_{n-1} \bigr\Vert \\ &\leq \bigl\Vert (I-\xi D)u_{n}-(I-\xi D)u_{n-1} \bigr\Vert = \Vert u_{n}-u_{n-1} \Vert . \end{aligned}$$

Note that $M:=J^{B_{1}}_{\lambda_{1}}[I+\gamma A^{\ast}(U-I)A]$ is nonexpansive, we have

3.14 $$\begin{aligned} \Vert u_{n}-u_{n-1} \Vert &= \bigl\Vert J^{B_{1}}_{\lambda _{1}} \bigl[I+\gamma A^{\ast}(U-I)A \bigr]x_{n}-J^{B_{1}}_{\lambda_{1}} \bigl[I+\gamma A^{\ast}(U-I)A \bigr]x_{n-1} \bigr\Vert \\ & \leq \Vert x_{n}-x_{n-1} \Vert . \end{aligned}$$

Substituting (3.13) and (3.14) for (3.12), we have

3.15 $$ \Vert y_{n}-y_{n-1} \Vert \leq \Vert x_{n}-x_{n-1} \Vert +|\beta_{n}- \beta_{n-1}|M_{4}+\sum^{N} _{i=1} \bigl\vert \eta ^{(n)}_{i}- \eta^{(n-1)}_{i} \bigr\vert \Vert T_{i}v_{n-1} \Vert . $$

This together with (3.11) leads to

3.16 $$\begin{aligned} &\Vert x_{n+1}-x_{n} \Vert \\ &\quad \leq \biggl[1- \gamma_{n}-\alpha_{n}\mu \biggl(\eta-\frac {\alpha_{n}\mu K^{2}}{2(1-\gamma_{n})} \biggr) \biggr] \Biggl[ \Vert x_{n}-x_{n-1} \Vert +|\beta _{n}-\beta_{n-1}|M_{4} \\ & \qquad {} +\sum^{N} _{i=1} \bigl\vert \eta^{(n)}_{i}-\eta ^{(n-1)}_{i} \bigr\vert \Vert T_{i}v_{n-1} \Vert \Biggr]+(\gamma_{n}+ \alpha_{n}\tau L) \Vert x_{n}-x_{n-1} \Vert \\ &\qquad {} +|\alpha_{n}-\alpha_{n-1}|M_{2}+| \gamma_{n}-\gamma _{n-1}|M_{3} \\ &\quad \leq \biggl[1-\gamma_{n}-\alpha_{n}\mu \biggl(\eta- \frac{\alpha_{n}\mu K^{2}}{2(1-\gamma_{n})} \biggr) \biggr] \Vert x_{n}-x_{n-1} \Vert +(\gamma_{n}+\alpha _{n}\tau L) \Vert x_{n}-x_{n-1} \Vert \\ & \qquad {} +|\alpha_{n}-\alpha_{n-1}|M_{2}+| \gamma_{n}-\gamma _{n-1}|M_{3}+| \beta_{n}-\beta_{n-1}|M_{4}+\sum ^{N} _{i=1} \bigl\vert \eta^{(n)}_{i}- \eta^{(n-1)}_{i} \bigr\vert \Vert T_{i}v_{n-1} \Vert \\ &\quad = \biggl[1-\alpha_{n} \biggl(\mu\eta-\frac{\alpha_{n}\mu K^{2}}{2(1-\gamma _{n})}-\tau L \biggr) \biggr] \Vert x_{n}-x_{n-1} \Vert +| \alpha_{n}- \alpha_{n-1}|M_{2} \\ &\qquad {} +|\gamma_{n}-\gamma_{n-1}|M_{3}+| \beta_{n}-\beta _{n-1}|M_{4}+\sum ^{N} _{i=1} \bigl\vert \eta^{(n)}_{i}- \eta ^{(n-1)}_{i} \bigr\vert \Vert T_{i}v_{n-1} \Vert . \end{aligned}$$

Noticing condition (v) and applying Lemma 2.5 to (3.16), we obtain

3.17 $$ \lim_{n\rightarrow\infty}\| x_{n+1}-x_{n} \|=0. $$

This completes the proof. □

### Lemma 3.2

*Let* $H_{1}$ *and* $H_{2}$ *be two real Hilbert spaces and* *C* *be a nonempty closed convex subset of* $H_{1}$. *Let* $A: H_{1}\rightarrow H_{2}$ *be a bounded linear operator*, $A^{\ast}$ *be the adjoint of* *A*, *and* *r* *be the spectral radius of the operator* $A^{\ast}A$. *Let* $f: H_{2}\rightarrow H_{2}$ *be a* *ρ*-*inverse strongly monotone operator and* $B_{1}: C\rightarrow2^{H_{1}}$, $B_{2}: H_{2}\rightarrow2^{H_{2}}$ *be two multi*-*valued maximal monotone operators*. *Let* $D: C\rightarrow H_{1}$ *be a* *δ*-*inverse strongly monotone operator*. *Assume that* $\{T_{i}\}^{N}_{i=1}: C\rightarrow C$ *is a finite family of* $k_{i}$-*strict pseudo*-*contraction mappings such that* $\mathcal{F}\neq \emptyset$. *Let* $P_{C}$ *be the metric projection of* $H_{1}$ *onto* *C*, *and* $F: C\rightarrow H_{1}$ *be an* *L*-*Lipschitzian mapping with constant* $L\geq0$. *Suppose that* $V: C\rightarrow H_{1}$ *is an* *η*-*strongly monotone and* *K*-*Lipschitzian mapping*, *where* *η* *and* *μ* *satisfy the conditions of Lemma *3.1. *For* $x_{1}\in C$, *let* $\{ x_{n}\}$ *be a sequence of* *C* *generated by* (1.7). *Assume that conditions* (i)*–*(v) *in Lemma *3.1 *hold*. *Then* $\{x_{n}\}$ *converges strongly to* $q\in\mathcal{F}$, *which solves the following variational inequality*:

$$ \langle\mu Vq-\tau Fq, q-p\rangle\leq 0, \quad \forall p\in\mathcal{F}. $$

### Proof

The proof of the lemma is divided into four steps.

*Step* 1. We claim $\lim_{n\rightarrow\infty}\| x_{n}-G_{n}x_{n}\|=0$.

Indeed, take $\forall p\in\mathcal{F}$. From (3.1) and (3.6), we have

3.18 $$\begin{aligned} & \Vert x_{n+1}-p \Vert ^{2} \\ &\quad = \bigl\Vert P_{C} \bigl[\alpha_{n}\tau Fx_{n}+\gamma_{n}x_{n}+ \bigl((1-\gamma _{n})I-\alpha_{n}\mu V \bigr)y_{n} \bigr]-p \bigr\Vert ^{2} \\ &\quad \leq \bigl\Vert \alpha_{n}\tau Fx_{n}+ \gamma_{n}x_{n}+ \bigl((1-\gamma _{n})I- \alpha_{n}\mu V \bigr)y_{n}-p \bigr\Vert ^{2} \\ &\quad = \bigl\Vert \alpha_{n}(\tau Fx_{n}-\mu Vp)+ \gamma_{n}(x_{n}-p)+ \bigl[(1-\gamma _{n})I- \alpha_{n}\mu V \bigr]y_{n}- \bigl[(1-\gamma_{n})I- \alpha_{n}\mu V \bigr]p \bigr\Vert ^{2} \\ &\quad \leq \bigl\Vert \alpha_{n}(\tau Fx_{n}-\mu Vp)+ \bigl[(1-\gamma_{n})I-\alpha _{n}\mu V \bigr]y_{n}- \bigl[(1-\gamma_{n})I-\alpha_{n}\mu V \bigr]p \bigr\Vert ^{2} \\ & \qquad {} +\gamma_{n}^{2} \Vert x_{n}-p \Vert ^{2}+2\gamma_{n} \Vert x_{n}-p \Vert \bigl\Vert \alpha _{n}(\tau Fx_{n}-\mu Vp) \\ & \qquad {} + \bigl[(1-\gamma_{n})I-\alpha_{n}\mu V \bigr]y_{n}- \bigl[(1-\gamma_{n})I-\alpha _{n}\mu V \bigr]p \bigr\Vert \\ &\quad \leq \biggl[1-\gamma_{n}-\alpha_{n}\mu \biggl(\eta- \frac{\alpha_{n}\mu K^{2}}{2(1-\gamma_{n})} \biggr) \biggr]^{2} \Vert y_{n}-p \Vert ^{2}+\alpha_{n}^{2} \Vert \tau Fx_{n}-\mu Vp \Vert ^{2} \\ & \qquad {} +2\alpha_{n} \biggl[1-\gamma_{n}- \alpha_{n}\mu \biggl(\eta-\frac{\alpha _{n}\mu K^{2}}{2(1-\gamma_{n})} \biggr) \biggr] \Vert \tau Fx_{n}-\mu Vp \Vert \Vert y_{n}-p \Vert \\ & \qquad {}+ \gamma_{n}^{2} \Vert x_{n}-p \Vert ^{2} +2\gamma_{n} \Vert x_{n}-p \Vert \bigl\{ \alpha_{n} \Vert \tau Fx_{n}-\mu Vp \Vert \\ &\qquad {}+ \bigl\Vert \bigl[(1-\gamma_{n})I-\alpha_{n}\mu V \bigr]y_{n}- \bigl[(1-\gamma_{n})I-\alpha _{n}\mu V \bigr]p \bigr\Vert \bigr\} \\ &\quad \leq \biggl[1-\gamma_{n}-\alpha_{n}\mu \biggl(\eta- \frac{\alpha_{n}\mu K^{2}}{2(1-\gamma_{n})} \biggr) \biggr]^{2} \Vert u_{n}-p \Vert ^{2}+\alpha_{n}^{2} \Vert \tau Fx_{n}-\mu Vp \Vert ^{2} \\ & \qquad {} +2\alpha_{n} \Vert \tau Fx_{n}-\mu Vp \Vert \Vert y_{n}-p \Vert +\gamma_{n}^{2} \Vert x_{n}-p \Vert ^{2} \\ & \qquad {} +2\gamma_{n} \Vert x_{n}-p \Vert \biggl\{ \alpha_{n} \Vert \tau Fx_{n}-\mu Vp \Vert + \biggl[1- \gamma_{n}-\alpha_{n}\mu \biggl(\eta-\frac{\alpha_{n}\mu K^{2}}{2(1-\gamma_{n})} \biggr) \biggr] \Vert y_{n}-p \Vert \biggr\} \\ &\quad \leq \biggl[1-\gamma_{n}-\alpha_{n}\mu \biggl(\eta- \frac{\alpha_{n}\mu K^{2}}{2(1-\gamma_{n})} \biggr) \biggr]^{2} \bigl[ \Vert x_{n}-p \Vert ^{2}+\gamma(r\gamma-1) \bigl\Vert (U-I)Ax_{n} \bigr\Vert ^{2} \bigr] \\ & \qquad {} +\alpha_{n}^{2} \Vert \tau Fx_{n}- \mu Vp \Vert ^{2}+2\alpha_{n} \Vert \tau Fx_{n}-\mu Vp \Vert \Vert y_{n}-p \Vert + \gamma_{n}^{2} \Vert x_{n}-p \Vert ^{2} \\ & \qquad {} +2\gamma_{n} \Vert x_{n}-p \Vert \bigl( \alpha_{n} \Vert \tau Fx_{n}-\mu Vp \Vert + \Vert y_{n}-p \Vert \bigr), \end{aligned}$$

which implies

$$\begin{aligned} & \biggl[1-\gamma_{n}-\alpha_{n}\mu \biggl(\eta- \frac{\alpha_{n}\mu K^{2}}{2(1-\gamma_{n})} \biggr) \biggr]^{2}\gamma(1-r\gamma) \bigl\Vert (U-I)Ax_{n} \bigr\Vert ^{2} \\ &\quad \leq \biggl[1-\gamma_{n}-\alpha_{n}\mu \biggl(\eta- \frac{\alpha_{n}\mu K^{2}}{2(1-\gamma_{n})} \biggr) \biggr]^{2} \Vert x_{n}-p \Vert ^{2}+\alpha_{n}^{2} \Vert \tau Fx_{n}-\mu Vp \Vert ^{2} \\ &\qquad {}+2\alpha_{n} \Vert \tau Fx_{n}-\mu Vp \Vert \Vert y_{n}-p \Vert +\gamma_{n}^{2} \Vert x_{n}-p \Vert ^{2} \\ &\qquad {}+2\gamma_{n} \Vert x_{n}-p \Vert \bigl(\alpha _{n} \Vert \tau Fx_{n}-\mu Vp \Vert + \Vert y_{n}-p \Vert \bigr)- \Vert x_{n+1}-p \Vert ^{2} \\ &\quad \leq \Vert x_{n}-p \Vert ^{2}+ \biggl[ \gamma_{n}+\alpha_{n}\mu \biggl(\eta-\frac{\alpha _{n}\mu K^{2}}{2(1-\gamma_{n})} \biggr) \biggr]^{2} \Vert x_{n}-p \Vert ^{2}+ \alpha_{n}^{2} \Vert \tau Fx_{n}-\mu Vp \Vert ^{2} \\ & \qquad {} +2\alpha_{n} \Vert \tau Fx_{n}-\mu Vp \Vert \Vert y_{n}-p \Vert +\gamma_{n}^{2} \Vert x_{n}-p \Vert ^{2}+2\gamma_{n} \Vert x_{n}-p \Vert \bigl(\alpha_{n} \Vert \tau Fx_{n}-\mu Vp \Vert \\ & \qquad {} + \Vert y_{n}-p \Vert \bigr)- \Vert x_{n+1}-p \Vert ^{2} \\ &\quad \leq \biggl[\gamma_{n}+\alpha_{n}\mu \biggl(\eta- \frac{\alpha_{n}\mu K^{2}}{2(1-\gamma_{n})} \biggr) \biggr]^{2} \Vert x_{n}-p \Vert ^{2}+\alpha_{n}^{2} \Vert \tau Fx_{n}-\mu Vp \Vert ^{2} \\ & \qquad {} +2\alpha_{n} \Vert \tau Fx_{n}-\mu Vp \Vert \Vert y_{n}-p \Vert +\gamma_{n}^{2} \Vert x_{n}-p \Vert ^{2} \\ &\qquad {}+2\gamma_{n} \Vert x_{n}-p \Vert \bigl(\alpha _{n} \Vert \tau Fx_{n}-\mu Vp \Vert + \Vert y_{n}-p \Vert \bigr) \\ & \qquad {} + \Vert x_{n}-x_{n+1} \Vert \bigl( \Vert x_{n}-p \Vert + \Vert x_{n+1}-p \Vert \bigr). \end{aligned}$$

Since $\gamma(1-r\gamma)>0$, $\lim_{n\rightarrow\infty}\alpha _{n}=0$, $\lim_{n\rightarrow\infty}\gamma_{n}=0$, and $\{x_{n}\}$, $\{ y_{n}\}$ are bounded, from (3.17) we get

3.19 $$ \lim_{n\rightarrow\infty} \bigl\Vert (U-I)Ax_{n} \bigr\Vert =0. $$

In addition, by the firm nonexpansion of $J^{B_{1}}_{\lambda_{1}}$, (3.2), (3.6), and $\gamma\in(0,\frac{1}{r})$, we estimate

$$\begin{aligned} \Vert u_{n}-p \Vert ^{2}&= \bigl\Vert J^{B_{1}}_{\lambda_{1}} \bigl[x_{n}+\gamma A^{\ast }(U-I)Ax_{n} \bigr]-J^{B_{1}}_{\lambda_{1}}p \bigr\Vert ^{2} \\ &\leq \bigl\langle J^{B_{1}}_{\lambda_{1}} \bigl[x_{n}+\gamma A^{\ast }(U-I)Ax_{n} \bigr]-J^{B_{1}}_{\lambda_{1}}p, x_{n}+\gamma A^{\ast }(U-I)Ax_{n}-p \bigr\rangle \\ &= \bigl\langle u_{n}-p, x_{n}+\gamma A^{\ast}(U-I)Ax_{n}-p \bigr\rangle \\ &=\frac{1}{2} \bigl( \Vert u_{n}-p \Vert ^{2}+ \bigl\Vert x_{n}+\gamma A^{\ast}(U-I)Ax_{n}-p \bigr\Vert ^{2} \\ &\quad {}- \bigl\Vert (u_{n}-p)- \bigl[x_{n}+ \gamma A^{\ast}(U-I)Ax_{n}-p \bigr] \bigr\Vert ^{2} \bigr) \\ &\leq\frac{1}{2} \bigl[ \Vert u_{n}-p \Vert ^{2}+ \Vert x_{n}-p \Vert ^{2}+\gamma(r \gamma-1) \bigl\Vert (U-I)Ax_{n} \bigr\Vert ^{2} \\ &\quad {}- \bigl\Vert u_{n}-x_{n}-\gamma A^{\ast}(U-I)Ax_{n} \bigr\Vert ^{2} \bigr] \\ &\leq\frac{1}{2} \bigl[ \Vert u_{n}-p \Vert ^{2}+ \Vert x_{n}-p \Vert ^{2}- \bigl\Vert u_{n}-x_{n}-\gamma A^{\ast}(U-I)Ax_{n} \bigr\Vert ^{2} \bigr] \\ &=\frac{1}{2} \bigl[ \Vert u_{n}-p \Vert ^{2}+ \Vert x_{n}-p \Vert ^{2}- \Vert u_{n}-x_{n} \Vert ^{2}-\gamma^{2} \bigl\Vert A^{\ast}(U-I)Ax_{n} \bigr\Vert ^{2} \\ &\quad {}+2\gamma \bigl\langle u_{n}-x_{n}, A^{\ast}(U-I)Ax_{n} \bigr\rangle \bigr] \\ &\leq\frac{1}{2} \bigl[ \Vert u_{n}-p \Vert ^{2}+ \Vert x_{n}-p \Vert ^{2}- \Vert u_{n}-x_{n} \Vert ^{2}+2\gamma \bigl\langle u_{n}-x_{n}, A^{\ast}(U-I)Ax_{n} \bigr\rangle \bigr] \\ &=\frac{1}{2} \bigl[ \Vert u_{n}-p \Vert ^{2}+ \Vert x_{n}-p \Vert ^{2}- \Vert u_{n}-x_{n} \Vert ^{2}+2\gamma \bigl\langle A(u_{n}-x_{n}), (U-I)Ax_{n} \bigr\rangle \bigr] \\ &\leq\frac{1}{2} \bigl[ \Vert u_{n}-p \Vert ^{2}+ \Vert x_{n}-p \Vert ^{2}- \Vert u_{n}-x_{n} \Vert ^{2}+2\gamma \bigl\Vert A(u_{n}-x_{n}) \bigr\Vert \bigl\Vert (U-I)Ax_{n} \bigr\Vert \bigr], \end{aligned}$$

and hence

3.20 $$ \|u_{n}-p\|^{2}\leq\|x_{n}-p\|^{2}-\| u_{n}-x_{n}\|^{2}+2\gamma \bigl\Vert A(u_{n}-x_{n}) \bigr\Vert \bigl\Vert (U-I)Ax_{n} \bigr\Vert . $$

In view of (3.18) and (3.20), we obtain

$$\begin{aligned} & \Vert x_{n+1}-p \Vert ^{2} \\ &\quad \leq \biggl[1-\gamma_{n}-\alpha_{n}\mu \biggl(\eta- \frac{\alpha_{n}\mu K^{2}}{2(1-\gamma_{n})} \biggr) \biggr]^{2} \Vert u_{n}-p \Vert ^{2}+\alpha_{n}^{2} \Vert \tau Fx_{n}-\mu Vp \Vert ^{2} \\ & \qquad {} +2\alpha_{n} \Vert \tau Fx_{n}-\mu Vp \Vert \Vert y_{n}-p \Vert +\gamma_{n}^{2} \Vert x_{n}-p \Vert ^{2} \\ & \qquad {} +2\gamma_{n} \Vert x_{n}-p \Vert \biggl\{ \alpha_{n} \Vert \tau Fx_{n}-\mu Vp \Vert + \biggl[1- \gamma_{n}-\alpha_{n}\mu \biggl(\eta-\frac{\alpha_{n}\mu K^{2}}{2(1-\gamma_{n})} \biggr) \biggr] \Vert y_{n}-p \Vert \biggr\} \\ &\quad \leq \biggl[1-\gamma_{n}-\alpha_{n}\mu \biggl(\eta- \frac{\alpha_{n}\mu K^{2}}{2(1-\gamma_{n})} \biggr) \biggr]^{2} \Vert u_{n}-p \Vert ^{2}+\alpha_{n}^{2} \Vert \tau Fx_{n}-\mu Vp \Vert ^{2} \\ & \qquad {} +2\alpha_{n} \Vert \tau Fx_{n}-\mu Vp \Vert \Vert y_{n}-p \Vert +\gamma_{n}^{2} \Vert x_{n}-p \Vert ^{2} \\ &\qquad {}+2\gamma_{n} \Vert x_{n}-p \Vert \bigl(\alpha_{n} \Vert \tau Fx_{n}-\mu Vp \Vert + \Vert y_{n}-p \Vert \bigr) \\ &\quad \leq \biggl[1-\gamma_{n}-\alpha_{n}\mu \biggl(\eta- \frac{\alpha_{n}\mu K^{2}}{2(1-\gamma_{n})} \biggr) \biggr]^{2} \bigl[ \Vert x_{n}-p \Vert ^{2}- \Vert u_{n}-x_{n} \Vert ^{2} \\ &\qquad {}+2\gamma \bigl\Vert A(u_{n}-x_{n}) \bigr\Vert \bigl\Vert (U-I)Ax_{n} \bigr\Vert \bigr]+\alpha_{n}^{2} \Vert \tau Fx_{n}- \mu Vp \Vert ^{2} \\ & \qquad {}+2\alpha_{n} \Vert \tau Fx_{n}-\mu Vp \Vert \Vert y_{n}-p \Vert + \gamma_{n}^{2} \Vert x_{n}-p \Vert ^{2} \\ & \qquad {} +2\gamma_{n} \Vert x_{n}-p \Vert \bigl( \alpha_{n} \Vert \tau Fx_{n}-\mu Vp \Vert + \Vert y_{n}-p \Vert \bigr) \\ &\quad \leq \Vert x_{n}-p \Vert ^{2}+ \biggl[ \gamma_{n}+\alpha_{n}\mu \biggl(\eta-\frac{\alpha _{n}\mu K^{2}}{2(1-\gamma_{n})} \biggr) \biggr]^{2} \Vert x_{n}-p \Vert ^{2} \\ &\qquad {}- \biggl[1-\gamma _{n}-\alpha_{n}\mu \biggl(\eta- \frac{\alpha_{n}\mu K^{2}}{2(1-\gamma _{n})} \biggr) \biggr]^{2} \Vert u_{n}-x_{n} \Vert ^{2} \\ & \qquad {} +2\gamma \biggl[1-\gamma_{n}-\alpha_{n}\mu \biggl(\eta-\frac{\alpha _{n}\mu K^{2}}{2(1-\gamma_{n})} \biggr) \biggr]^{2} \bigl\Vert A(u_{n}-x_{n}) \bigr\Vert \bigl\Vert (U-I)Ax_{n} \bigr\Vert \\ & \qquad {}+\alpha_{n}^{2} \Vert \tau Fx_{n}-Vp \Vert ^{2} +2\alpha_{n} \Vert \tau Fx_{n}-\mu Vp \Vert \Vert y_{n}-p \Vert +\gamma_{n}^{2} \Vert x_{n}-p \Vert ^{2} \\ &\qquad {}+2\gamma_{n} \Vert x_{n}-p \Vert \bigl(\alpha_{n} \Vert \tau Fx_{n}-\mu Vp \Vert + \Vert y_{n}-p \Vert \bigr), \end{aligned}$$

which hence implies that

3.21 $$\begin{aligned} & \biggl[1-\gamma_{n}-\alpha_{n}\mu \biggl(\eta- \frac{\alpha_{n}\mu K^{2}}{2(1-\gamma_{n})} \biggr) \biggr]^{2} \Vert u_{n}-x_{n} \Vert ^{2} \\ &\quad \leq \Vert x_{n}-p \Vert ^{2}- \Vert x_{n+1}-p \Vert ^{2}+ \biggl[\gamma_{n}+ \alpha_{n}\mu \biggl(\eta-\frac{\alpha_{n}\mu K^{2}}{2(1-\gamma_{n})} \biggr) \biggr]^{2} \Vert x_{n}-p \Vert ^{2} \\ & \qquad {} +2\gamma \biggl[1-\gamma_{n}-\alpha_{n}\mu \biggl(\eta-\frac{\alpha _{n}\mu K^{2}}{2(1-\gamma_{n})} \biggr) \biggr]^{2} \bigl\Vert A(u_{n}-x_{n}) \bigr\Vert \bigl\Vert (U-I)Ax_{n} \bigr\Vert \\ & \qquad {} +\alpha_{n}^{2} \Vert \tau Fx_{n}- \mu Vp \Vert ^{2}+2\alpha_{n} \Vert \tau Fx_{n}-\mu Vp \Vert \Vert y_{n}-p \Vert + \gamma_{n}^{2} \Vert x_{n}-p \Vert ^{2} \\ & \qquad {} +2\gamma_{n} \Vert x_{n}-p \Vert \bigl( \alpha_{n} \Vert \tau Fx_{n}-\mu Vp \Vert + \Vert y_{n}-p \Vert \bigr) \\ &\quad \leq \Vert x_{n}-x_{n+1} \Vert \bigl( \Vert x_{n}-p \Vert + \Vert x_{n+1}-p \Vert \bigr)+ \biggl[ \gamma_{n}+\alpha _{n}\mu \biggl(\eta-\frac{\alpha_{n}\mu K^{2}}{2(1-\gamma_{n})} \biggr) \biggr]^{2} \Vert x_{n}-p \Vert ^{2} \\ &\qquad {} +2\gamma \biggl[1-\gamma_{n}-\alpha_{n}\mu \biggl(\eta-\frac{\alpha _{n}\mu K^{2}}{2(1-\gamma_{n})} \biggr) \biggr]^{2} \bigl\Vert A(u_{n}-x_{n}) \bigr\Vert \bigl\Vert (U-I)Ax_{n} \bigr\Vert \\ &\qquad {} +\alpha_{n}^{2} \Vert \tau Fx_{n}- \mu Vp \Vert ^{2}+2\alpha_{n} \Vert \tau Fx_{n}-\mu Vp \Vert \Vert y_{n}-p \Vert + \gamma_{n}^{2} \Vert x_{n}-p \Vert ^{2} \\ & \qquad {} +2\gamma_{n} \Vert x_{n}-p \Vert \bigl( \alpha_{n} \Vert \tau Fx_{n}-\mu Vp \Vert + \Vert y_{n}-p \Vert \bigr). \end{aligned}$$

From conditions (ii), (iv), (3.17), and (3.19), we get

3.22 $$ \lim_{n\rightarrow\infty}\| u_{n}-x_{n} \|=0. $$

According to (3.1) and (3.10), we obtain

$$\begin{aligned} \Vert x_{n+1}-p \Vert ^{2}&= \bigl\Vert P_{C} \bigl[\alpha_{n}\tau Fx_{n}+\gamma _{n}x_{n}+ \bigl((1-\gamma_{n})I- \alpha_{n}\mu V \bigr)y_{n} \bigr]-p \bigr\Vert ^{2} \\ &\leq \bigl\Vert \alpha_{n}\tau Fx_{n}+ \gamma_{n}x_{n}+ \bigl((1-\gamma _{n})I- \alpha_{n}\mu V \bigr)y_{n}-p \bigr\Vert ^{2} \\ &= \bigl\Vert \alpha_{n}(\tau Fx_{n}-\mu Vy_{n})+\gamma _{n}(x_{n}-y_{n})+y_{n}-p \bigr\Vert ^{2} \\ &= \Vert y_{n}-p \Vert ^{2}+ \bigl\Vert \alpha_{n}(\tau Fx_{n}-\mu Vy_{n})+\gamma _{n}(x_{n}-y_{n}) \bigr\Vert ^{2} \\ & \quad {} +2 \bigl\langle \alpha_{n}(\tau Fx_{n}-\mu Vy_{n})+\gamma _{n}(x_{n}-y_{n}), y_{n}-p \bigr\rangle \\ &\leq \Vert v_{n}-p \Vert ^{2}+ \bigl\Vert \alpha_{n}(\tau Fx_{n}-\mu Vy_{n})+\gamma _{n}(x_{n}-y_{n}) \bigr\Vert ^{2} \\ & \quad {} +2 \bigl\langle \alpha_{n}(\tau Fx_{n}-\mu Vy_{n})+\gamma _{n}(x_{n}-y_{n}), y_{n}-p \bigr\rangle \\ &\leq \Vert u_{n}-p \Vert ^{2}+\xi(\xi-2\delta) \Vert Du_{n}-Dp \Vert ^{2}+ \bigl\Vert \alpha _{n}( \tau Fx_{n}-\mu Vy_{n})+\gamma_{n}(x_{n}-y_{n}) \bigr\Vert ^{2} \\ & \quad {} +2 \bigl\langle \alpha_{n}(\tau Fx_{n}-\mu Vy_{n})+\gamma _{n}(x_{n}-y_{n}), y_{n}-p \bigr\rangle \\ &\leq \Vert x_{n}-p \Vert ^{2}+\xi(\xi-2\delta) \Vert Du_{n}-Dp \Vert ^{2}+ \bigl\Vert \alpha _{n}( \tau Fx_{n}-\mu Vy_{n})+\gamma_{n}(x_{n}-y_{n}) \bigr\Vert ^{2} \\ & \quad {} +2 \bigl\langle \alpha_{n}(\tau Fx_{n}-\mu Vy_{n})+\gamma _{n}(x_{n}-y_{n}), y_{n}-p \bigr\rangle , \end{aligned}$$

and hence

$$\begin{aligned} &\xi(2\delta-\xi) \Vert Du_{n}-Dp \Vert ^{2} \\ &\quad \leq \Vert x_{n}-p \Vert ^{2}- \Vert x_{n+1}-p \Vert ^{2}+ \bigl\Vert \alpha_{n}( \tau Fx_{n}-\mu V y_{n})+\gamma_{n}(x_{n}-y_{n}) \bigr\Vert ^{2} \\ &\qquad {} +2 \bigl\langle \alpha_{n}(\tau Fx_{n}-\mu Vy_{n})+\gamma _{n}(x_{n}-y_{n}), y_{n}-p \bigr\rangle \\ &\quad \leq \Vert x_{n}-x_{n+1} \Vert \bigl( \Vert x_{n}-p \Vert + \Vert x_{n+1}-p \Vert \bigr)+ \bigl\Vert \alpha_{n}(\tau Fx_{n}-\mu Vy_{n})+ \gamma_{n}(x_{n}-y_{n}) \bigr\Vert ^{2} \\ &\qquad {} +2 \bigl\langle \alpha_{n}(\tau Fx_{n}-\mu Vy_{n})+\gamma _{n}(x_{n}-y_{n}), y_{n}-p \bigr\rangle \\ &\quad \leq \Vert x_{n}-x_{n+1} \Vert \bigl( \Vert x_{n}-p \Vert + \Vert x_{n+1}-p \Vert \bigr)+ \bigl( \alpha_{n} \Vert \tau Fx_{n}-\mu Vy_{n} \Vert +\gamma_{n} \Vert x_{n}-y_{n} \Vert \bigr)^{2} \\ & \qquad {} +2 \bigl(\alpha_{n} \Vert \tau Fx_{n}-\mu Vy_{n} \Vert +\gamma_{n} \Vert x_{n}-y_{n} \Vert \bigr) \Vert y_{n}-p \Vert . \end{aligned}$$

Since $\lim_{n\rightarrow\infty}\alpha_{n}=0$, $\lim_{n\rightarrow \infty}\gamma_{n}=0$, and $\{x_{n}\}$, $\{y_{n}\}$ are bounded, by (3.17), we obtain

3.23 $$ \lim_{n\rightarrow\infty}\| Du_{n}-Dp\|=0. $$

It follows from (2.9), (3.1), and (3.10) that

$$\begin{aligned} &\Vert v_{n}-p \Vert ^{2} \\ &\quad = \bigl\Vert P_{C}(I-\xi D)u_{n}-P_{C}(I-\xi D)p \bigr\Vert ^{2} \\ &\quad \leq \bigl\langle P_{C}(I-\xi D)u_{n}-P_{C}(I- \xi D)p, (I-\xi D)u_{n}-(I-\xi D)p \bigr\rangle \\ &\quad = \bigl\langle v_{n}-p, (I-\xi D)u_{n}-(I-\xi D)p \bigr\rangle \\ &\quad =\frac{1}{2} \bigl\{ \Vert v_{n}-p \Vert ^{2}+ \bigl\Vert u_{n}-p-\xi(Du_{n}-Dp) \bigr\Vert ^{2} \\ &\qquad {}- \bigl\Vert (v_{n}-p)- \bigl[(I-\xi D)u_{n}-(I-\xi D)p \bigr] \bigr\Vert ^{2} \bigr\} \\ &\quad \leq\frac{1}{2} \bigl\{ \Vert v_{n}-p \Vert ^{2}+ \Vert u_{n}-p \Vert ^{2}+\xi(\xi-2\delta ) \Vert Du_{n}-Dp \Vert ^{2}- \bigl\Vert (v_{n}-u_{n})+\xi(Du_{n}-Dp) \bigr\Vert ^{2} \bigr\} \\ &\quad \leq\frac{1}{2} \bigl\{ \Vert v_{n}-p \Vert ^{2}+ \Vert u_{n}-p \Vert ^{2}+\xi(\xi-2\delta ) \Vert Du_{n}-Dp \Vert ^{2} \\ & \qquad {} - \Vert v_{n}-u_{n} \Vert ^{2}- \xi^{2} \Vert Du_{n}-Dp \Vert ^{2}-2\xi\langle v_{n}-u_{n}, Du_{n}-Dp\rangle \bigr\} \\ &\quad =\frac{1}{2} \bigl\{ \Vert v_{n}-p \Vert ^{2}+ \Vert u_{n}-p \Vert ^{2}-2\xi\delta \Vert Du_{n}-Dp \Vert ^{2} \\ & \qquad {} - \Vert v_{n}-u_{n} \Vert ^{2}+2 \xi\langle u_{n}-v_{n}, Du_{n}-Dp\rangle \bigr\} \\ &\quad \leq\frac{1}{2} \bigl( \Vert v_{n}-p \Vert ^{2}+ \Vert u_{n}-p \Vert ^{2}- \Vert v_{n}-u_{n} \Vert ^{2}+2\xi\langle u_{n}-v_{n}, Du_{n}-Dp\rangle \bigr) \\ &\quad \leq\frac{1}{2} \bigl( \Vert v_{n}-p \Vert ^{2}+ \Vert x_{n}-p \Vert ^{2}- \Vert v_{n}-u_{n} \Vert ^{2}+2\xi \Vert u_{n}-v_{n} \Vert \Vert Du_{n}-Dp \Vert \bigr), \end{aligned}$$

which implies

3.24 $$ \|v_{n}-p\|^{2}\leq\|x_{n}-p\|^{2}-\| v_{n}-u_{n}\|^{2}+2\xi\|u_{n}-v_{n} \|\|Du_{n}-Dp\|. $$

From (3.18) and (3.24), we have

$$\begin{aligned} & \Vert x_{n+1}-p \Vert ^{2} \\ &\quad \leq \biggl[1-\gamma_{n}-\alpha_{n}\mu \biggl(\eta- \frac{\alpha_{n}\mu K^{2}}{2(1-\gamma_{n})} \biggr) \biggr]^{2} \Vert y_{n}-p \Vert ^{2}+\alpha_{n}^{2} \Vert \tau Fx_{n}-\mu Vp \Vert ^{2} \\ & \qquad {} +2\alpha_{n} \biggl[1-\gamma_{n}- \alpha_{n}\mu \biggl(\eta-\frac{\alpha _{n}\mu K^{2}}{2(1-\gamma_{n})} \biggr) \biggr] \Vert \tau Fx_{n}-\mu Vp \Vert \Vert y_{n}-p \Vert \\ & \qquad {} + \gamma_{n}^{2} \Vert x_{n}-p \Vert ^{2}+2\gamma_{n} \Vert x_{n}-p \Vert \bigl\{ \alpha_{n} \Vert \tau Fx_{n}-\mu Vp \Vert \\ &\qquad {}+ \bigl\Vert \bigl[(1-\gamma_{n})I-\alpha_{n}\mu V \bigr]y_{n}- \bigl[(1-\gamma_{n})I-\alpha _{n}\mu V \bigr]p \bigr\Vert \bigr\} \\ &\quad \leq \biggl[1-\gamma_{n}-\alpha_{n}\mu \biggl(\eta- \frac{\alpha_{n}\mu K^{2}}{2(1-\gamma_{n})} \biggr) \biggr]^{2} \Vert v_{n}-p \Vert ^{2}+\alpha_{n}^{2} \Vert \tau Fx_{n}-\mu Vp \Vert ^{2} \\ & \qquad {} +2\alpha_{n} \Vert \tau Fx_{n}-\mu Vp \Vert \Vert y_{n}-p \Vert +\gamma_{n}^{2} \Vert x_{n}-p \Vert ^{2} \\ & \qquad {} +2\gamma_{n} \Vert x_{n}-p \Vert \biggl\{ \alpha_{n} \Vert \tau Fx_{n}-\mu Vp \Vert + \biggl[1- \gamma_{n}-\alpha_{n}\mu \biggl(\eta-\frac{\alpha_{n}\mu K^{2}}{2(1-\gamma_{n})} \biggr) \biggr] \Vert y_{n}-p \Vert \biggr\} \\ &\quad \leq \biggl[1-\gamma_{n}-\alpha_{n}\mu \biggl(\eta- \frac{\alpha_{n}\mu K^{2}}{2(1-\gamma_{n})} \biggr) \biggr]^{2} \\ &\qquad {}\times\bigl( \Vert x_{n}-p \Vert ^{2}- \Vert v_{n}-u_{n} \Vert ^{2}+2\xi \Vert u_{n}-v_{n} \Vert \Vert Du_{n}-Dp \Vert \bigr) \\ & \qquad {} +\alpha_{n}^{2} \Vert \tau Fx_{n}- \mu Vp \Vert ^{2}+2\alpha_{n} \Vert \tau Fx_{n}-\mu Vp \Vert \Vert y_{n}-p \Vert + \gamma_{n}^{2} \Vert x_{n}-p \Vert ^{2} \\ & \qquad {} +2\gamma_{n} \Vert x_{n}-p \Vert \bigl( \alpha_{n} \Vert \tau Fx_{n}-\mu Vp \Vert + \Vert y_{n}-p \Vert \bigr) \\ &\quad \leq \Vert x_{n}-p \Vert ^{2}+ \biggl[ \gamma_{n}+\alpha_{n}\mu \biggl(\eta-\frac{\alpha _{n}\mu K^{2}}{2(1-\gamma_{n})} \biggr) \biggr]^{2} \Vert x_{n}-p \Vert ^{2} \\ &\qquad {}- \biggl[1-\gamma _{n}-\alpha_{n}\mu \biggl(\eta- \frac{\alpha_{n}\mu K^{2}}{2(1-\gamma _{n})} \biggr) \biggr]^{2} \Vert v_{n}-u_{n} \Vert ^{2} \\ & \qquad {} +2\xi \Vert u_{n}-v_{n} \Vert \Vert Du_{n}-Dp \Vert +\alpha_{n}^{2} \Vert \tau Fx_{n}-\mu Vp \Vert ^{2}+2\alpha_{n} \Vert \tau Fx_{n}-\mu Vp \Vert \Vert y_{n}-p \Vert \\ & \qquad {} +\gamma_{n}^{2} \Vert x_{n}-p \Vert ^{2}+2\gamma_{n} \Vert x_{n}-p \Vert \bigl(\alpha _{n} \Vert \tau Fx_{n}-\mu Vp \Vert + \Vert y_{n}-p \Vert \bigr), \end{aligned}$$

and hence

$$\begin{aligned} & \biggl[1-\gamma_{n}-\alpha_{n}\mu \biggl(\eta- \frac{\alpha_{n}\mu K^{2}}{2(1-\gamma_{n})} \biggr) \biggr]^{2} \Vert v_{n}-u_{n} \Vert ^{2} \\ &\quad \leq \Vert x_{n}-p \Vert ^{2}- \Vert x_{n+1}-p \Vert ^{2}+ \biggl[\gamma_{n}+ \alpha_{n}\mu \biggl(\eta-\frac{\alpha_{n}\mu K^{2}}{2(1-\gamma_{n})} \biggr) \biggr]^{2} \Vert x_{n}-p \Vert ^{2} \\ &\qquad {} +2\xi \Vert u_{n}-v_{n} \Vert \Vert Du_{n}-Dp \Vert +\alpha_{n}^{2} \Vert \tau Fx_{n}-\mu Vp \Vert ^{2}+2\alpha_{n} \Vert \tau Fx_{n}-\mu Vp \Vert \Vert y_{n}-p \Vert \\ & \qquad {} +\gamma_{n}^{2} \Vert x_{n}-p \Vert ^{2}+2\gamma_{n} \Vert x_{n}-p \Vert \bigl(\alpha _{n} \Vert \tau Fx_{n}-\mu Vp \Vert + \Vert y_{n}-p \Vert \bigr) \\ &\quad \leq \Vert x_{n}-x_{n+1} \Vert \bigl( \Vert x_{n}-p \Vert - \Vert x_{n+1}-p \Vert \bigr)+ \biggl[ \gamma_{n}+\alpha _{n}\mu \biggl(\eta-\frac{\alpha_{n}\mu K^{2}}{2(1-\gamma_{n})} \biggr) \biggr]^{2} \Vert x_{n}-p \Vert ^{2} \\ &\qquad {} +2\xi \Vert u_{n}-v_{n} \Vert \Vert Du_{n}-Dp \Vert +\alpha_{n}^{2} \Vert \tau Fx_{n}-\mu Vp \Vert ^{2}+2\alpha_{n} \Vert \tau Fx_{n}-\mu Vp \Vert \Vert y_{n}-p \Vert \\ &\qquad {} +\gamma_{n}^{2} \Vert x_{n}-p \Vert ^{2}+2\gamma_{n} \Vert x_{n}-p \Vert \bigl(\alpha _{n} \Vert \tau Fx_{n}-\mu Vp \Vert + \Vert y_{n}-p \Vert \bigr). \end{aligned}$$

Since $\lim_{n\rightarrow\infty}\alpha_{n}=0$, $\lim_{n\rightarrow \infty}\gamma_{n}=0$, and $\{x_{n}\}$, $\{y_{n}\}$ are bounded, we obtain from (3.17) and (3.23)

3.25 $$ \lim_{n\rightarrow\infty}\| v_{n}-u_{n} \|=0. $$

Combining (3.22) with (3.25), we get

3.26 $$ \|v_{n}-x_{n}\|\leq\|v_{n}-u_{n}\|+ \| u_{n}-x_{n}\|\rightarrow0 \quad \mbox{as } n\rightarrow \infty. $$

By (3.1) and the nonexpansion of $S_{n}$, we obtain

$$\begin{aligned} \Vert x_{n}-S_{n}x_{n} \Vert &\leq \Vert x_{n}-x_{n+1} \Vert + \Vert x_{n+1}-S_{n}x_{n} \Vert \\ &\leq \Vert x_{n}-x_{n+1} \Vert + \bigl\Vert \alpha_{n}\tau Fx_{n}+\gamma _{n}x_{n}+ \bigl((1-\gamma_{n})I-\alpha_{n}\mu V \bigr)y_{n}-S_{n}x_{n} \bigr\Vert \\ &= \Vert x_{n}-x_{n+1} \Vert + \bigl\Vert \alpha_{n}(\tau Fx_{n}-\mu Vy_{n})+y_{n}-S_{n}x_{n}+ \gamma_{n}(x_{n}-y_{n}) \bigr\Vert \\ &\leq \Vert x_{n}-x_{n+1} \Vert +\alpha_{n} \Vert \tau Fx_{n}-\mu Vy_{n} \Vert + \Vert S_{n}v_{n}-S_{n}x_{n} \Vert + \gamma_{n} \Vert x_{n}-y_{n} \Vert \\ &\leq \Vert x_{n}-x_{n+1} \Vert +\alpha_{n} \Vert \tau Fx_{n}-\mu Vy_{n} \Vert + \Vert v_{n}-x_{n} \Vert +\gamma_{n} \Vert x_{n}-y_{n} \Vert . \end{aligned}$$

It follows from $\lim_{n\rightarrow\infty}\alpha_{n}=0$, $\lim_{n\rightarrow\infty}\gamma_{n}=0$, (3.17) and (3.26) that

3.27 $$ \lim_{n\rightarrow\infty}\| x_{n}-S_{n}x_{n} \|=0. $$

In the meantime, observe that

$$\begin{aligned} \Vert x_{n}-S_{n}x_{n} \Vert &= \bigl\Vert \beta_{n}x_{n}+(1-\beta_{n})G_{n}x_{n}-x_{n} \bigr\Vert \\ &= \bigl\Vert \beta_{n}x_{n}+(1-\beta_{n})G_{n}x_{n}- \beta_{n}x_{n}-(1-\beta _{n})x_{n} \bigr\Vert \\ &=(1-\beta_{n}) \Vert x_{n}-G_{n}x_{n} \Vert . \end{aligned}$$

From condition (iii), we have

3.28 $$ \lim_{n\rightarrow\infty}\| x_{n}-G_{n}x_{n} \|=0. $$

*Step* 2. We claim that $q\in\mathcal{F}$, for *q* any weak cluster point of $\{x_{n}\}$.

Indeed, by condition (v), we know that $\lim_{n\rightarrow\infty }\eta_{i}^{(n)}=\eta_{i}$ for every $1\leq i\leq N$. It is easy to see that each $\eta_{i}>0$ and $\sum^{N}_{i=1}\eta_{i}=1$. Define $G:=\sum^{N}_{i=1}\eta_{i}T_{i}$. Then it follows from Lemma 2.3 that $G: C\rightarrow C$ is a *k*-strict pseudo-contraction and $F(G)=F(G_{n})=\bigcap^{N}_{i=1}F(T_{i})$. Furthermore, $G_{n}x\rightarrow Gx$ as $n\rightarrow\infty$ for all $x\in C$. In addition, $S: C\rightarrow C$ is defined as $Sx:=lx+(1-l)Gx$. Then *S* is nonexpansive and $F(S)=F(G)$ by Lemma 2.1. Observe that

$$\begin{aligned} \Vert x_{n}-Sx_{n} \Vert &\leq \Vert x_{n}-S_{n}x_{n} \Vert + \Vert S_{n}x_{n}-Sx_{n} \Vert \\ &= \Vert x_{n}-S_{n}x_{n} \Vert + \bigl\Vert \beta_{n}x_{n}+(1-\beta _{n})G_{n}x_{n}-lx_{n}-(1-l)Gx_{n} \bigr\Vert \\ &\leq \Vert x_{n}-S_{n}x_{n} \Vert +| \beta_{n}-l| \Vert x_{n}-G_{n}x_{n} \Vert +(1-\beta _{n}) \Vert G_{n}x_{n}-Gx_{n} \Vert \\ &\leq \Vert x_{n}-S_{n}x_{n} \Vert +| \beta_{n}-l| \Vert x_{n}-G_{n}x_{n} \Vert +(1-\beta _{n})\sum^{N} _{i=1} \bigl\vert \eta^{(n)}_{i}- \eta_{i} \bigr\vert \Vert T_{i}x_{n} \Vert . \end{aligned}$$

From (3.27) and (3.28), we obtain

3.29 $$ \lim_{n\rightarrow\infty}\| x_{n}-Sx_{n} \|=0. $$

Since $\{x_{n}\}$ is bounded, we may assume that *q* is any weak cluster point of $\{x_{n}\}$. Hence, there exists a subsequence $\{ x_{n_{k}}\}$ of $\{x_{n}\}$, which converges weakly to *q*. Now, since *S* is nonexpansive, by (3.29) and Lemma 2.6, we obtain that $q\in F(S)$. Thus, we have $q\in F(G)=F(G_{n})=\bigcap^{N}_{i=1}F(T_{i})$.

In addition, we rewrite $u_{n_{k}}=J^{B_{1}}_{\lambda _{1}}[x_{n_{k}}+\gamma A^{\ast}(U-I)Ax_{n_{k}}]$ as

3.30 $$ \frac{x_{n_{k}}-u_{n_{k}}+\gamma A^{\ast }(U-I)Ax_{n_{k}}}{\lambda_{1}}\in B_{1}u_{n_{k}}. $$

Letting $k\rightarrow\infty$ in (3.30) and using (3.19), (3.22) and the fact that the graph of a maximal monotone operator is weakly-strongly closed, we have $0\in B_{1}q$, i.e., $q\in \operatorname{SOLVIP}(B_{1})$. Furthermore, since $x_{n}$ and $u_{n}$ have the same asymptotical behavior, $Ax_{n_{k}}$ weakly converges to *Aq*. It follows from (3.19), the nonexpansion of *U*, and Lemma 2.6 that $(I-U)Aq=0$. Thus, by Proposition 2.3, we have $0\in f(Aq)+B_{2}(Aq)$, i.e., $Aq\in \operatorname{SOLVIP}(B_{2})$. As a result, $q\in\varGamma$.

Moreover, it follows from (3.25) that $v_{n_{k}}$ weakly converges to *q*. Define

$$ \mathcal{H}v= \textstyle\begin{cases} Dv+N_{C}v, & v\in C, \\ \emptyset, & v\in H_{1}\setminus C. \end{cases} $$

Then $\mathcal{H}$ is maximal monotone by Proposition 2.5. Take $\forall(v, w)\in\operatorname{Graph}(\mathcal{H})$. It is easy to see that $w-Dv\in N_{C}v$. Since $v_{n}\in C$, we have

3.31 $$ \langle v-v_{n}, w-Dv\rangle\geq0. $$

Combining (2.10) with $v_{n}=P_{C}(u_{n}-\xi Du_{n})$, we get

3.32 $$ \langle u_{n}-\xi Du_{n}-v_{n}, v_{n}-v\rangle\geq0, $$

and hence

3.33 $$ \biggl\langle v-v_{n}, \frac{v_{n}-u_{n}}{\xi }+Du_{n} \biggr\rangle \geq0. $$

Thus, from (3.31) and (3.33), we obtain

$$\begin{aligned} \langle v-v_{n_{k}}, w\rangle&\geq\langle v-v_{n_{k}}, Dv\rangle \\ &\geq\langle v-v_{n_{k}}, Dv\rangle- \biggl\langle v-v_{n_{k}}, Du_{n_{k}}+\frac{v_{n_{k}}-u_{n_{k}}}{\xi} \biggr\rangle \\ &=\langle v-v_{n_{k}}, Dv-Dv_{n_{k}}\rangle+\langle v-v_{n_{k}}, Dv_{n_{k}}-Du_{n_{k}}\rangle- \biggl\langle v-v_{n_{k}}, \frac {v_{n_{k}}-u_{n_{k}}}{\xi} \biggr\rangle \\ &\geq\delta\|Dv-Dv_{n_{k}}\|^{2}+\langle v-v_{n_{k}}, Dv_{n_{k}}-Du_{n_{k}}\rangle- \biggl\langle v-v_{n_{k}}, \frac {v_{n_{k}}-u_{n_{k}}}{\xi} \biggr\rangle \\ &\geq\langle v-v_{n_{k}}, Dv_{n_{k}}-Du_{n_{k}}\rangle- \biggl\langle v-v_{n_{k}}, \frac{v_{n_{k}}-u_{n_{k}}}{\xi} \biggr\rangle . \end{aligned}$$

Letting $k\rightarrow\infty$, we have $\langle v-q, w\rangle\geq0$ as $k\rightarrow\infty$. Since $\mathcal{H}$ is maximal monotone, we get $q\in\mathcal{H}^{-1}0$. So it follows from Proposition 2.5 that $q\in \operatorname{VI}(C, D)$. Therefore, $q\in\bigcap^{N}_{i=1}F(T_{i})\cap \varGamma\cap \operatorname{VI}(C, D)=\mathcal{F}$.

*Step* 3. We claim that

$$ \limsup_{n\rightarrow\infty } \bigl\langle (\mu V-\tau F)q, q-x_{n} \bigr\rangle \leq0, $$

where $q=\lim_{t\rightarrow0}x_{t}$ with $x_{t}$ being the fixed point of the contraction $\varPsi_{t}$ on *C* defined by

$$ \varPsi_{t}x:=P_{C} \bigl[t\tau Fx+(I-t\mu V)Tx \bigr], \quad \forall x\in C, $$

here $t\in(0, 2\eta/K^{2})$ and $Tx:=SP_{C}(I-\xi D)J^{B_{1}}_{\lambda}[I+\gamma A^{\ast}(U-I)A]x$, $\forall x\in C$.

Indeed, first, for each $x, y\in C$, note that

$$\begin{aligned} &\Vert Tx-Ty \Vert \\ &\quad = \bigl\Vert SP_{C}(I-\xi D)J^{B_{1}}_{\lambda_{1}} \bigl[I+\gamma A^{\ast }(U-I)A \bigr]x-SP_{C}(I-\xi D)J^{B_{1}}_{\lambda_{1}} \bigl[I+\gamma A^{\ast }(U-I)A \bigr]y \bigr\Vert \\ &\quad \leq \bigl\Vert P_{C}(I-\xi D)J^{B_{1}}_{\lambda_{1}} \bigl[I+\gamma A^{\ast }(U-I)A \bigr]x-P_{C}(I-\xi D)J^{B_{1}}_{\lambda_{1}} \bigl[I+\gamma A^{\ast }(U-I)A \bigr]y \bigr\Vert \\ &\quad \leq \bigl\Vert (I-\xi D)J^{B_{1}}_{\lambda_{1}} \bigl[I+\gamma A^{\ast }(U-I)A \bigr]x-(I-\xi D)J^{B_{1}}_{\lambda_{1}} \bigl[I+ \gamma A^{\ast}(U-I)A \bigr]y \bigr\Vert \\ &\quad \leq \bigl\Vert J^{B_{1}}_{\lambda_{1}} \bigl[I+\gamma A^{\ast }(U-I)A \bigr]x-J^{B_{1}}_{\lambda_{1}} \bigl[I+\gamma A^{\ast}(U-I)A \bigr]y \bigr\Vert \\ &\quad \leq \Vert x-y \Vert , \end{aligned}$$

which implies that *T* is nonexpansive. Further, we estimate

$$\begin{aligned} &\Vert Tx_{n}-x_{n} \Vert \\ &\quad = \bigl\Vert SP_{C}(I-\xi D)J^{B_{1}}_{\lambda_{1}} \bigl[I+\gamma A^{\ast}(U-I)A \bigr]x_{n}-x_{n} \bigr\Vert \\ &\quad = \bigl\Vert SP_{C}(I-\xi D)u_{n}-x_{n} \bigr\Vert \\ &\quad = \Vert Sv_{n}-x_{n} \Vert \\ &\quad \leq \Vert Sv_{n}-S_{n}v_{n} \Vert + \Vert S_{n}v_{n}-x_{n} \Vert \\ &\quad = \bigl\Vert \beta_{n}v_{n}+(1-\beta_{n})G_{n}v_{n}-lv_{n}-(1-l)Gv_{n} \bigr\Vert + \Vert S_{n}v_{n}-S_{n}x_{n}+S_{n}x_{n}-x_{n} \Vert \\ &\quad \leq|\beta_{n}-l| \Vert v_{n}-Gv_{n} \Vert +(1-\beta_{n}) \Vert G_{n}v_{n}-Gv_{n} \Vert + \Vert S_{n}v_{n}-S_{n}x_{n} \Vert + \Vert S_{n}x_{n}-x_{n} \Vert \\ &\quad \leq|\beta_{n}-l| \Vert v_{n}-Gv_{n} \Vert +(1-\beta_{n})\sum^{N} _{i=1}\bigl| \eta^{(n)}_{i}-\eta_{i}\bigr| \Vert T_{i}v_{n} \Vert + \Vert v_{n}-x_{n} \Vert + \Vert S_{n}x_{n}-x_{n} \Vert . \end{aligned}$$

From condition (iii), (3.26), and (3.27), we obtain

3.34 $$ \lim_{n\rightarrow\infty}\| Tx_{n}-x_{n} \|=0. $$

Also, for each $x, y\in C$, it follows from Lemma 2.8 that $\varPsi_{t}$ has a unique fixed point $x_{t}\in C$ such that $x_{t}=P_{C}[t\tau Fx+(I-t\mu V)Tx_{t}]$, and the net $\{x_{t}\}_{t\in(0, 1)}$ converges strongly as $t\rightarrow0$ to a fixed point *q* of *T* which solves the variational inequality $\langle(\mu V-\tau F)q, q-p\rangle\leq 0$, $\forall p\in F(T)$.

Next, from the above arguments, we know that $F(S)\cap\varGamma\cap \operatorname{VI}(C, D)=\bigcap^{N}_{i=1}F(T_{i})\cap\varGamma\cap \operatorname{VI}(C, D)=\mathcal {F}$. Further, for $\forall q_{1}\in F(T)=F(SP_{C}(I-\xi D)J^{B_{1}}_{\lambda_{1}}[I+\gamma A^{\ast}(U-I)A])$ and $\forall q_{2}\in F(S)\cap\varGamma\cap \operatorname{VI}(C, D)$. Then we have $q_{2}=J^{B_{1}}_{\lambda_{1}}q_{2}$, $Aq_{2}=UAq_{2}$, $q_{2}=J^{B_{1}}_{\lambda_{1}}[I+\gamma A^{\ast}(U-I)A]q_{2}$, and $q_{2}=P_{C}(I-\xi D)q_{2}$. By the nonexpansion of *S*, $P_{C}(I-\xi D)$ and $J^{B_{1}}_{\lambda_{1}}$, we get

$$\begin{aligned} &\Vert q_{1}-q_{2} \Vert ^{2} \\ &\quad = \bigl\Vert SP_{C}(I-\xi D)J^{B_{1}}_{\lambda_{1}} \bigl[I+\gamma A^{\ast}(U-I)A \bigr]q_{1}-SP_{C}(I-\xi D)J^{B_{1}}_{\lambda_{1}} \bigl[I+\gamma A^{\ast}(U-I)A \bigr]q_{2} \bigr\Vert ^{2} \\ &\quad \leq \bigl\Vert P_{C}(I-\xi D)J^{B_{1}}_{\lambda_{1}} \bigl[I+\gamma A^{\ast }(U-I)A \bigr]q_{1}-P_{C}(I-\xi D)J^{B_{1}}_{\lambda_{1}} \bigl[I+\gamma A^{\ast }(U-I)A \bigr]q_{2} \bigr\Vert ^{2} \\ &\quad \leq \bigl\Vert J^{B_{1}}_{\lambda_{1}} \bigl[I+\gamma A^{\ast }(U-I)A \bigr]q_{1}-J^{B_{1}}_{\lambda_{1}} \bigl[I+\gamma A^{\ast}(U-I)A \bigr]q_{2} \bigr\Vert ^{2} \\ &\quad \leq \bigl\Vert \bigl[I+\gamma A^{\ast}(U-I)A \bigr]q_{1}- \bigl[I+\gamma A^{\ast }(U-I)A \bigr]q_{2} \bigr\Vert ^{2} \\ &\quad = \bigl\Vert q_{1}+\gamma A^{\ast}(U-I)Aq_{1}-q_{2} \bigr\Vert ^{2} \\ &\quad \leq \Vert q_{1}-q_{2} \Vert ^{2}+\gamma(r \gamma-1) \bigl\Vert (U-I)Aq_{1} \bigr\Vert ^{2}. \end{aligned}$$

Since $\gamma\in(0, \frac{1}{r})$, we infer that

3.35 $$ (U-I)Aq_{1}=0, $$

it follows from Proposition 2.3 that $Aq_{1}\in \operatorname{SOLVIP}(B_{2})$. In addition, since $J^{B_{1}}_{\lambda_{1}}$ is firmly nonexpansive, from (3.35) we estimate

$$\begin{aligned} &\Vert q_{1}-q_{2} \Vert ^{2} \\ &\quad \leq \bigl\Vert J^{B_{1}}_{\lambda_{1}} \bigl[I+\gamma A^{\ast }(U-I)A \bigr]q_{1}-J^{B_{1}}_{\lambda_{1}} \bigl[I+\gamma A^{\ast}(U-I)A \bigr]q_{2} \bigr\Vert ^{2} \\ &\quad \leq \bigl\langle J^{B_{1}}_{\lambda_{1}} \bigl[I+\gamma A^{\ast }(U-I)A \bigr]q_{1}-J^{B_{1}}_{\lambda_{1}} \bigl[I+\gamma A^{\ast}(U-I)A \bigr]q_{2}, \\ &\qquad \bigl[I+\gamma A^{\ast}(U-I)A \bigr]q_{1}- \bigl[I+\gamma A^{\ast}(U-I)A \bigr]q_{2} \bigr\rangle \\ &\quad = \bigl\langle J^{B_{1}}_{\lambda_{1}} \bigl[I+\gamma A^{\ast }(U-I)A \bigr]q_{1}-q_{2}, \bigl[I+\gamma A^{\ast}(U-I)A \bigr]q_{1}- \bigl[I+\gamma A^{\ast }(U-I)A \bigr]q_{2} \bigr\rangle \\ &\quad = \bigl\langle J^{B_{1}}_{\lambda_{1}}q_{1}-q_{2}, q_{1}-q_{2} \bigr\rangle \leq \bigl\Vert J^{B_{1}}_{\lambda_{1}}q_{1}-q_{2} \bigr\Vert \Vert q_{1}-q_{2} \Vert \\ &\quad = \bigl\Vert J^{B_{1}}_{\lambda_{1}}q_{1}-J^{B_{1}}_{\lambda_{1}}q_{2} \bigr\Vert \Vert q_{1}-q_{2} \Vert \leq \Vert q_{1}-q_{2} \Vert ^{2}, \end{aligned}$$

which implies

3.36 $$ \|q_{1}-q_{2}\|^{2}= \bigl\langle J^{B_{1}}_{\lambda_{1}}q_{1}-q_{2}, q_{1}-q_{2} \bigr\rangle , $$

hence

3.37 $$ \bigl\langle J^{B_{1}}_{\lambda_{1}}q_{1}-q_{1}, q_{1}-q_{2} \bigr\rangle =0. $$

Meanwhile, by (3.35) and (3.37), we have

$$\begin{aligned} \Vert q_{1}-q_{2} \Vert ^{2}&\geq \bigl\Vert J^{B_{1}}_{\lambda_{1}} \bigl[I+\gamma A^{\ast }(U-I)A \bigr]q_{1}-J^{B_{1}}_{\lambda_{1}} \bigl[I+\gamma A^{\ast}(U-I)A \bigr]q_{2} \bigr\Vert ^{2} \\ &= \bigl\Vert J^{B_{1}}_{\lambda_{1}} \bigl[I+\gamma A^{\ast}(U-I)A \bigr]q_{1}-q_{2} \bigr\Vert ^{2} \\ &= \bigl\Vert J^{B_{1}}_{\lambda_{1}}q_{1}-q_{1}+q_{1}-q_{2} \bigr\Vert ^{2} \\ &= \bigl\Vert J^{B_{1}}_{\lambda_{1}}q_{1}-q_{1} \bigr\Vert ^{2}+ \Vert q_{1}-q_{2} \Vert ^{2}+2 \bigl\langle J^{B_{1}}_{\lambda_{1}}q_{1}-q_{1}, q_{1}-q_{2} \bigr\rangle \\ &= \bigl\Vert J^{B_{1}}_{\lambda_{1}}q_{1}-q_{1} \bigr\Vert ^{2}+ \Vert q_{1}-q_{2} \Vert ^{2}, \end{aligned}$$

and hence $J^{B_{1}}_{\lambda_{1}}q_{1}=q_{1}$. Thus, $0\in B_{1}q_{1}$, i.e., $q_{1}\in \operatorname{SOLVIP}(B_{1})$. As a result, we get $q_{1}\in\varGamma$. By the assumption $q_{1}=Tq_{1}=SP_{C}(I-\xi D)J^{B_{1}}_{\lambda_{1}}[I+\gamma A^{\ast}(U-I)A]q_{1}$, we have $q_{1}=SP_{C}(I-\xi D)q_{1}$. Moreover, from the above arguments, we get

$$\begin{aligned} \Vert q_{1}-q_{2} \Vert ^{2}&= \bigl\Vert SP_{C}(I-\xi D)q_{1}-SP_{C}(I-\xi D)q_{2} \bigr\Vert ^{2} \\ &\leq \bigl\Vert P_{C}(I-\xi D)q_{1}-P_{C}(I- \xi D)q_{2} \bigr\Vert ^{2} \\ &\leq \bigl\Vert (I-\xi D)q_{1}-(I-\xi D)q_{2} \bigr\Vert ^{2} \\ &= \bigl\Vert q_{1}-q_{2}-\xi(Dq_{1}-Dq_{2}) \bigr\Vert ^{2} \\ &\leq \Vert q_{1}-q_{2} \Vert ^{2}+\xi(\xi-2 \delta) \Vert Dq_{1}-Dq_{2} \Vert ^{2} \\ &\leq \Vert q_{1}-q_{2} \Vert ^{2}, \end{aligned}$$

thus, we have

3.38 $$ Dq_{1}-Dq_{2}=0. $$

From (3.38), we obtain

$$\begin{aligned} \Vert q_{1}-q_{2} \Vert ^{2}&= \bigl\Vert SP_{C}(I-\xi D)q_{1}-SP_{C}(I-\xi D)q_{2} \bigr\Vert ^{2} \\ &\leq \bigl\Vert P_{C}(I-\xi D)q_{1}-P_{C}(I- \xi D)q_{2} \bigr\Vert ^{2} \\ &\leq \bigl\langle P_{C}(I-\xi D)q_{1}-P_{C}(I- \xi D)q_{2}, (I-\xi D)q_{1}-(I-\xi D)q_{2} \bigr\rangle \\ &= \bigl\langle P_{C}(I-\xi D)q_{1}-q_{2}, q_{1}-q_{2}-\xi (Dq_{1}-Dq_{2}) \bigr\rangle \\ &= \bigl\langle P_{C}(I-\xi D)q_{1}-q_{2}, q_{1}-q_{2} \bigr\rangle \\ &\leq \bigl\Vert P_{C}(I-\xi D)q_{1}-q_{2} \bigr\Vert \Vert q_{1}-q_{2} \Vert \\ &= \bigl\Vert P_{C}(I-\xi D)q_{1}-P_{C}(I-\xi D)q_{2} \bigr\Vert \Vert q_{1}-q_{2} \Vert \\ &\leq \Vert q_{1}-q_{2} \Vert ^{2}, \end{aligned}$$

and hence

3.39 $$ \|q_{1}-q_{2}\|^{2}= \bigl\langle P_{C}(I-\xi D)q_{1}-q_{2}, q_{1}-q_{2} \bigr\rangle , $$

that is,

3.40 $$ \bigl\langle P_{C}(I-\xi D)q_{1}-q_{1}, q_{1}-q_{2} \bigr\rangle =0. $$

Meanwhile, from (3.40), we get

$$\begin{aligned} \Vert q_{1}-q_{2} \Vert ^{2}&\geq \bigl\Vert P_{C}(I-\xi D)q_{1}-P_{C}(I-\xi D)q_{2} \bigr\Vert ^{2} \\ &= \bigl\Vert P_{C}(I-\xi D)q_{1}-q_{2} \bigr\Vert ^{2} \\ &= \bigl\Vert P_{C}(I-\xi D)q_{1}-q_{1}+q_{1}-q_{2} \bigr\Vert ^{2} \\ &= \bigl\Vert P_{C}(I-\xi D)q_{1}-q_{1} \bigr\Vert ^{2}+ \Vert q_{1}-q_{2} \Vert ^{2}+2 \bigl\langle P_{C}(I-\xi D)q_{1}-q_{1}, q_{1}-q_{2} \bigr\rangle \\ &= \bigl\Vert P_{C}(I-\xi D)q_{1}-q_{1} \bigr\Vert ^{2}+ \Vert q_{1}-q_{2} \Vert ^{2} \\ &\geq \Vert q_{1}-q_{2} \Vert ^{2}, \end{aligned}$$

which immediately implies $P_{C}(I-\xi D)q_{1}=q_{1}$, and so $q_{1}\in \operatorname{VI}(C, D)$. It follows from $q_{1}=SP_{C}(I-\xi D)q_{1}$ that $q_{1}=Sq_{1}$, i.e., $q_{1}\in F(S)$. Thus, $q_{1}\in F(S)\cap \operatorname{VI}(C, D)$. Since $q_{1}\in\varGamma$, we obtain that $q_{1}\in F(S)\cap\varGamma \cap \operatorname{VI}(C, D)$, which implies that $F(T)\subset F(S)\cap\varGamma\cap \operatorname{VI}(C, D)$. In addition, it is easy to see that $F(S)\cap\varGamma\cap \operatorname{VI}(C, D)\subset F(T)$. Therefore, $F(T)=F(S)\cap\varGamma\cap \operatorname{VI}(C, D)=\bigcap^{N}_{i=1}F(T_{i})\cap\varGamma\cap \operatorname{VI}(C, D)=\mathcal{F}$.

Finally, we take a subsequence $\{x_{n_{k}}\}$ of $\{x_{n}\}$, assume that $x_{n_{k}}\rightharpoonup\omega$, where $\omega\in F(T)=\mathcal{F}$. By using Lemma 2.6 and (3.34), we have

$$ \limsup_{n\rightarrow\infty } \bigl\langle (\mu V-\tau F)q, q-x_{n} \bigr\rangle =\limsup_{k\rightarrow\infty} \bigl\langle (\mu V-\tau F)q, q-x_{n_{k}} \bigr\rangle = \bigl\langle (\mu V-\tau F)q, q-\omega \bigr\rangle \leq0. $$

*Step* 4. We claim $\lim_{n\rightarrow\infty}\|x_{n}-q\|=0$.

Indeed, we put

3.41 $$ z_{n}=\alpha_{n}\tau Fx_{n}+\gamma _{n}x_{n}+ \bigl[(1-\gamma_{n})I- \alpha_{n}\mu V \bigr]y_{n}. $$

From (2.10), (3.1), (3.9), and (3.41), we obtain

$$\begin{aligned} & \Vert x_{n+1}-q \Vert ^{2} \\ &\quad =\langle P_{C}z_{n}-z_{n}, x_{n+1}-q\rangle+\langle z_{n}-q, x_{n+1}-q\rangle \\ &\quad =\langle P_{C}z_{n}-z_{n}, P_{C}z_{n}-q\rangle+\langle z_{n}-q, x_{n+1}-q\rangle \\ &\quad \leq\langle z_{n}-q, x_{n+1}-q\rangle \\ &\quad = \bigl\langle \alpha_{n}\tau Fx_{n}+ \gamma_{n}x_{n}+ \bigl[(1-\gamma _{n})I- \alpha_{n}\mu V \bigr]y_{n}-q, x_{n+1}-q \bigr\rangle \\ &\quad = \bigl\langle \bigl[(1-\gamma_{n})I-\alpha_{n}\mu V \bigr]y_{n}- \bigl[(1-\gamma _{n})I-\alpha_{n} \mu V \bigr]q \\ &\qquad {} +\alpha_{n}(\tau Fx_{n}-\mu Vq)+ \gamma_{n}(x_{n}-q), x_{n+1}-q \bigr\rangle \\ &\quad = \bigl\langle \bigl[(1-\gamma_{n})I-\alpha_{n}\mu V \bigr]y_{n}- \bigl[(1-\gamma _{n})I-\alpha_{n} \mu V \bigr]q, x_{n+1}-q \bigr\rangle \\ & \qquad {} + \bigl\langle \alpha_{n}(\tau Fx_{n}-\tau Fq), x_{n+1}-q \bigr\rangle +\alpha_{n}\langle\tau Fq-\mu Vq, x_{n+1}-q\rangle+\gamma _{n}\langle x_{n}-q, x_{n+1}-q\rangle \\ &\quad \leq \bigl\Vert \bigl[(1-\gamma_{n})I-\alpha_{n} \mu V \bigr]y_{n}- \bigl[(1-\gamma _{n})I- \alpha_{n}\mu V \bigr]q \bigr\Vert \Vert x_{n+1}-q \Vert \\ &\qquad {} +\alpha_{n}\tau L \Vert x_{n}-q \Vert \Vert x_{n+1}-q \Vert +\alpha_{n}\langle\tau Fq-\mu Vq, x_{n+1}-q\rangle+\gamma_{n} \Vert x_{n}-q \Vert \Vert x_{n+1}-q \Vert \\ &\quad \leq \biggl[1-\gamma_{n}-\alpha_{n}\mu \biggl(\eta- \frac{\alpha_{n}\mu K^{2}}{2(1-\gamma_{n})} \biggr) \biggr] \Vert y_{n}-q \Vert \Vert x_{n+1}-q \Vert +\alpha_{n}\tau L \Vert x_{n}-q \Vert \Vert x_{n+1}-q \Vert \\ & \qquad {} +\alpha_{n}\langle\tau Fq-\mu Vq, x_{n+1}-q \rangle+\gamma_{n} \Vert x_{n}-q \Vert \Vert x_{n+1}-q \Vert \\ &\quad \leq \biggl[1-\gamma_{n}-\alpha_{n}\mu \biggl(\eta- \frac{\alpha_{n}\mu K^{2}}{2(1-\gamma_{n})} \biggr) \biggr] \Vert x_{n}-q \Vert \Vert x_{n+1}-q \Vert +\alpha_{n}\tau L \Vert x_{n}-q \Vert \Vert x_{n+1}-q \Vert \\ & \qquad {} +\alpha_{n}\langle\tau Fq-\mu Vq, x_{n+1}-q \rangle+\gamma_{n} \Vert x_{n}-q \Vert \Vert x_{n+1}-q \Vert \\ &\quad = \biggl[1-\alpha_{n} \biggl(\mu\eta-\frac{\alpha_{n}\mu K^{2}}{2(1-\gamma _{n})}-\tau L \biggr) \biggr] \Vert x_{n}-q \Vert \Vert x_{n+1}-q \Vert +\alpha_{n}\langle\tau Fq-\mu Vq, x_{n+1}-q\rangle \\ &\quad \leq\frac{1}{2} \biggl[1-\alpha_{n} \biggl(\mu\eta- \frac{\alpha_{n}\mu K^{2}}{2(1-\gamma_{n})}-\tau L \biggr) \biggr] \bigl( \Vert x_{n}-q \Vert ^{2}+ \Vert x_{n+1}-q \Vert ^{2} \bigr) \\ &\qquad {}+ \alpha_{n}\langle\tau Fq-\mu Vq, x_{n+1}-q\rangle \\ &\quad \leq\frac{1}{2} \biggl[1-\alpha_{n} \biggl(\mu\eta- \frac{\alpha_{n}\mu K^{2}}{2(1-\gamma_{n})}-\tau L \biggr) \biggr] \Vert x_{n}-q \Vert ^{2}+\frac{1}{2} \Vert x_{n+1}-q \Vert ^{2} \\ &\qquad {}+\alpha_{n}\langle\tau Fq-\mu Vq, x_{n+1}-q \rangle, \end{aligned}$$

which implies that

3.42 $$\begin{aligned} \|x_{n+1}-q\|^{2} \leq&\biggl[1-\alpha_{n}\biggl(\mu \eta -\frac{\alpha_{n}\mu K^{2}}{2(1-\gamma_{n})}-\tau L\biggr)\biggr]\|x_{n}-q\| ^{2} \\ &{}+2\alpha_{n}\langle\tau Fq-\mu Vq, x_{n+1}-q \rangle. \end{aligned}$$

Put $a_{n}=\alpha_{n}(\mu\eta-\frac{\alpha_{n}\mu K^{2}}{2(1-\gamma_{n})}-\tau L)$ and $c_{n}=\frac{2\langle\tau Fq-\mu Vq, x_{n+1}-q\rangle}{\mu\eta-\frac{\alpha_{n}\mu K^{2}}{2(1-\gamma_{n})}-\tau L}$. Applying Lemma 2.5 to (3.42), we obtain $\lim_{n\rightarrow\infty}\|x_{n}-q\|=0$. This completes the proof. □

### Theorem 3.1

*Let* $H_{1}$ *and* $H_{2}$ *be two real Hilbert spaces and* *C* *be a nonempty closed convex subset of* $H_{1}$. *Let* $A: H_{1}\rightarrow H_{2}$ *be a bounded linear operator*, $A^{\ast}$ *be the adjoint of* *A*, *and* *r* *be the spectral radius of the operator* $A^{\ast}A$. *Let* $f: H_{2}\rightarrow H_{2}$ *be a* *ρ*-*inverse strongly monotone operator and* $B_{1}: C\rightarrow2^{H_{1}}$, $B_{2}: H_{2}\rightarrow2^{H_{2}}$ *be two multi*-*valued maximal monotone operators*. *Let* $D: C\rightarrow H_{1}$ *be a* *δ*-*inverse strongly monotone operator*. *Assume that* $\{T_{i}\}^{N}_{i=1}: C\rightarrow C$ *is a finite family of* $k_{i}$-*strict pseudo*-*contraction mappings such that* $\mathcal{F}\neq \emptyset$. *Let* $P_{C}$ *be the metric projection of* $H_{1}$ *onto* *C*, *and* $F: C\rightarrow H_{1}$ *be an* *L*-*Lipschitzian mapping with constant* $L\geq0$. *Suppose that* $V: C\rightarrow H_{1}$ *is an* *η*-*strongly monotone and* *K*-*Lipschitzian mapping*, *where* *η* *and* *μ* *satisfy the conditions of Lemma *3.1. *For* $x_{1}\in C$, *let* $\{ x_{n}\}$ *be a sequence of* *C* *generated by* (1.7). *Assume that conditions* (i)*–*(v) *in Lemma *3.1 *hold*. *Then* $\{x_{n}\}$ *converges strongly to* $q\in\mathcal{F}$, *which solves the following variational inequality*:

$$ \langle\mu Vq-\tau Fq, q-p\rangle\leq 0, \quad \forall p\in\mathcal{F}. $$

### Proof

Combining the proof of Lemma 3.1 with the proof of Lemma 3.2, we can obtain the conclusion. □

### Remark 3.1

Compared with Theorem 3.1 of Jitsupa et al. [1], our result is different from it in the following aspects:

(i) We not only change the parameter *λ* of resolvent operators $J^{B_{1}}_{\lambda}$ and $J^{B_{2}}_{\lambda}$ into different parameters $\lambda_{1}$ and $\lambda_{2}$, but also change the resolvent operator $J^{B_{2}}_{\lambda}$ into $J^{B_{2}}_{\lambda _{2}}(I-\lambda_{2}f)$ which is more general than $J^{B_{2}}_{\lambda }$. It is worth stressing that the parameter *λ* of resolvent operators $J^{B_{1}}_{\lambda}$ and $J^{B_{2}}_{\lambda}$ in many results is the same *λ*; see, e.g., [1, 11–13]. Thus our result improves and extends these results and other related results.
(ii) We improve and extend Theorem 3.1 of Jitsupa et al. [1]. Especially, we use the Lipschitzian instead of the contraction, and also use the *η*-strongly monotone and *K*-Lipschitzian operator instead of the strong positive linear bounded operator to construct our iteration process.
(iii) It is worth mentioning here that our result in this paper is more applicable and efficient than the result of Jitsupa et al. [1]. We give the definite domains and ranges of $B_{1}$ and $B_{2}$ to make the iterative scheme (1.6) well-defined. We also modify the iterative scheme (1.6) by adding the projection operator. As a result, our result can be applied to finding a common solution of SMVIP (1.3) and VIP (2.7) and fixed point problem of a finite family of strict pseudo-contraction mappings instead of SVIP (1.2) and fixed point problem of a finite family of strict pseudo-contraction mappings.

In Theorem 3.1, if $\lambda_{1}=\lambda_{2}$, $f=D\equiv0$, $\gamma _{n}=0$, *F* is a contraction mapping, and *V* is a strongly positive bounded linear operator, then we get the following corollary immediately.

### Corollary 3.1

*Let* $H_{1}$ *and* $H_{2}$ *be two real Hilbert spaces and* *C* *be a nonempty closed convex subset of* $H_{1}$. *Let* $A: H_{1}\rightarrow H_{2}$ *be a bounded linear operator*, $A^{\ast }$ *be the adjoint of* *A*, *and* *r* *be the spectral radius of the operator* $A^{\ast}A$. *Let* $B_{1}: C\rightarrow2^{H_{1}}$, $B_{2}: H_{2}\rightarrow2^{H_{2}}$ *be two multi*-*valued maximal monotone operators*. *Assume that* $\{T_{i}\}^{N}_{i=1}: C\rightarrow C$ *is a finite family of* $k_{i}$-*strict pseudo*-*contraction mappings such that* $\mathcal{\widetilde{F}}:=\bigcap^{N}_{i=1}F(T_{i})\cap\varGamma\neq \emptyset$. *Let* $f: C\rightarrow H_{1}$ *be a contraction mapping with constant* $\rho\in(0, 1)$ *and* $D: C\rightarrow H_{1}$ *be a strongly positive bounded linear operator with coefficient* $\overline{\tau }>0$. *For* $x_{1}\in C$, *let* $\{x_{n}\}$ *be a sequence generated by the following scheme*:

$$\textstyle\begin{cases} u_{n}=J^{B_{1}}_{\lambda}[x_{n}+\gamma A^{\ast}(J^{B_{2}}_{\lambda }-I)Ax_{n}], \\ y_{n}=\beta_{n}u_{n}+(1-\beta_{n})\sum^{N} _{i=1}\eta _{i}^{(n)}T_{i}u_{n}, \\ x_{n+1}=\alpha_{n}\tau f(x_{n})+(I-\alpha_{n}D)y_{n}, \quad n\geq1. \end{cases} $$

*Assume that conditions* (ii), (iii) *in Lemma *3.1 *and the following conditions hold*:

(i) $\lambda>0$, $0<\gamma<\frac{1}{r}$;
(ii) $\sum^{N}_{i=1}\eta^{(n)}_{i}=1$, $\sum^{\infty}_{n=1}(|\alpha _{n+1}-\alpha_{n}|+|\beta_{n+1}-\beta_{n}|+\sum^{N}_{i=1}|\eta ^{(n+1)}_{i}-\eta^{(n)}_{i}|)<\infty$.

*Then the sequence* $\{x_{n}\}$ *converges strongly to* $q\in\mathcal {\widetilde{F}}$, *which solves the following variational inequality*:

$$ \langle Dq-\tau fq, q-p\rangle\leq 0, \quad \forall p\in\mathcal{\widetilde{F}}. $$

## Numerical examples

The purpose of this section is to give an example and numerical results to support Theorem 3.1.

### Example 4.1

Let $H_{1}=H_{2}=\mathbb{R}^{3}$ and $C=[0, +\infty)\times[0, +\infty)\times[0, +\infty)$. Let the inner product $\langle\cdot, \cdot\rangle: \mathbb{R}^{3}\times\mathbb{R}^{3}\rightarrow \mathbb{R}$ be defined by $\langle x, y\rangle=x\cdot y=x_{1}y_{1}+x_{2}y_{2}+x_{3}y_{3}$ and the usual norm $\|\cdot\|: \mathbb{R}^{3}\rightarrow\mathbb{R}$ be defined by $\|x\|=\sqrt {x_{1}^{2}+x_{2}^{2}+x_{3}^{2}}$. Let two operators of matrix multiplication $B_{1}: C\rightarrow\mathbb{R}^{3}$, $B_{2}: \mathbb {R}^{3}\rightarrow\mathbb{R}^{3}$ be defined by

$$B_{1}=\left[\begin{array}{ccc} 1 & 0 & 0\\ 0 & 2 & 0\\ 0 & 0 & 3 \end{array}\right]\;\text{and}\;B_{2}=\left[\begin{array}{ccc} 2 & 0 & 0\\ 0 & 5 & 0\\ 0 & 0 & 3 \end{array}\right].$$

Then we can define the resolvent operators $J^{B_{1}}_{\lambda_{1}}$ and $J^{B_{2}}_{\lambda_{2}}$ on $\mathbb{R}^{3}$ associated with $B_{1}$ and $B_{2}$ where $\lambda_{1}, \lambda_{2}>0$. Let

$$A=\left[\begin{array}{ccc} 1 & 0 & 0\\ 0 & 2 & 0\\ 0 & 0 & 1 \end{array}\right]\in R^{3\times 3}$$

be a singular matrix operator and $A^{\ast}$ be the adjoint of *A*. It is easy to calculate that

$$A^{*}=\left[\begin{array}{ccc} 2 & 0 & 0\\ 0 & 1 & 0\\ 0 & 0 & 2 \end{array}\right].$$

The mappings $T_{i}: C\rightarrow C$ defined by $T_{1}x=(\frac {x_{1}}{10(1+x_{1})}, \frac{x_{2}}{10(1+x_{2})}, \frac {x_{3}}{10(1+x_{3})})$, $T_{2}x=(\frac{|\sin x_{1}|}{20(1+x_{1})}, \frac{|\sin x_{2}|}{20(1+x_{2})}, \frac{|\sin x_{3}|}{20(1+x_{3})})$, and $T_{3}x=(\frac{x_{1}}{30+x_{1}}, \frac{x_{2}}{30+x_{2}}, \frac {x_{3}}{30+x_{3}})$ are $k_{i}$-strict pseudo-contractions for $i=1, 2, 3$ (see [29]). Let $fx=\frac{1}{2}x$ ($\forall x\in\mathbb{R}^{3}$), $Dx=\frac{1}{3}x$ ($\forall x\in C$), $Vx=\frac{1}{2}x$ ($\forall x\in C$), and $Fx=\frac{3}{2}x$ ($\forall x\in C$). Now, we present the following algorithm.

### Algorithm 4.2

*Step* 0.: Choose the initial point $x_{1}=(2, 3, 4)\in C$. Put $\lambda_{1}=\frac{1}{2}$, $\lambda_{2}=\frac{1}{3}$, $\gamma=\frac {1}{2}$, $\xi=\frac{1}{2}$, $\beta_{n}=\frac{1}{10}$, $\eta^{n}_{1}=\eta ^{n}_{2}=\eta^{n}_{3}=\frac{1}{3}$, $\alpha_{n}=\frac{1}{8n}$, $\tau =\frac{1}{6}$, $\gamma_{n}=\frac{1}{10n}$, $\mu=\frac{2}{3}$ which satisfy the all assumed conditions of Theorem 3.1, and let $n=1$.
*Step* 1.: Given $x_{n}\in C$, compute $x_{n+1}\in C$ as follows:
  $$\textstyle\begin{cases} u_{n}=J^{B_{1}}_{\frac{1}{2}}[x_{n}+\frac{1}{2} A^{\ast }(J^{B_{2}}_{\frac{1}{3}}(I-\frac{1}{3}f)-I)Ax_{n}], \\ v_{n}=P_{C}(u_{n}-\frac{1}{2}Du_{n}), \\ y_{n}=\frac{1}{10}v_{n}+\frac{9}{10}\sum^{3} _{i=1}\frac{1}{3}T_{i}v_{n}, \\ x_{n+1}=P_{C}[\frac{1}{8n}\frac{1}{6} Fx_{n}+\frac {1}{10n}x_{n}+((1-\frac{1}{10n})I-\frac{1}{8n}\frac{2}{3}V)y_{n}],\quad n\geq1. \end{cases} $$
*Step* 2.: Put $n:=n+1$ and go to Step 1.

Setting $\|x_{n+1}-x_{n}\|\leq10^{-8}$ as a stop criterion, we get the numerical results of Algorithm 4.2.

Table 1 shows the values of the components of sequence $x_{n}$ and $\| x_{n+1}-x_{n}\|$.

**Table 1:** Values of the components of $x_{n}$ and $\|x_{n+1}-x_{n}\|$

*n*	$x^{1}_{n}$	$x^{2}_{n}$	$x^{3}_{n}$	$\|x_{n+1}-x_{n}\|$
1	2.0000	3.0000	4.0000	4.5762
2	3.3463 × 10^−1^	4.4135 × 10^−1^	5.9093 × 10^−1^	6.8947 × 10^−1^
3	5.4164 × 10^−2^	6.4209 × 10^−2^	8.6473 × 10^−2^	1.0297 × 10^−1^
4	8.4481 × 10^−3^	9.1707 × 10^−3^	1.2416 × 10^−2^	1.5088 × 10^−2^
5	1.2664 × 10^−3^	1.2838 × 10^−3^	1.7454 × 10^−3^	2.1608 × 10^−3^
6	1.8212 × 10^−4^	1.7606 × 10^−4^	2.4004 × 10^−4^	3.0176 × 10^−4^
7	2.5079 × 10^−5^	2.3641 × 10^−5^	3.2281 × 10^−5^	4.1013 × 10^−5^
8	3.3006 × 10^−6^	3.1068 × 10^−6^	4.2426 × 10^−6^	5.4163 × 10^−6^
9	4.1426 × 10^−7^	3.9941 × 10^−7^	5.4467 × 10^−7^	6.9425 × 10^−7^
10	4.9466 × 10^−8^	5.0206 × 10^−8^	6.8263 × 10^−8^	8.6332 × 10^−8^
11	5.6050 × 10^−9^	6.1673 × 10^−9^	8.3472 × 10^−9^	1.0419 × 10^−8^
12	6.0092 × 10^−10^	7.3997 × 10^−10^	9.9524 × 10^−10^	1.2216 × 10^−9^

**Figure 1:**
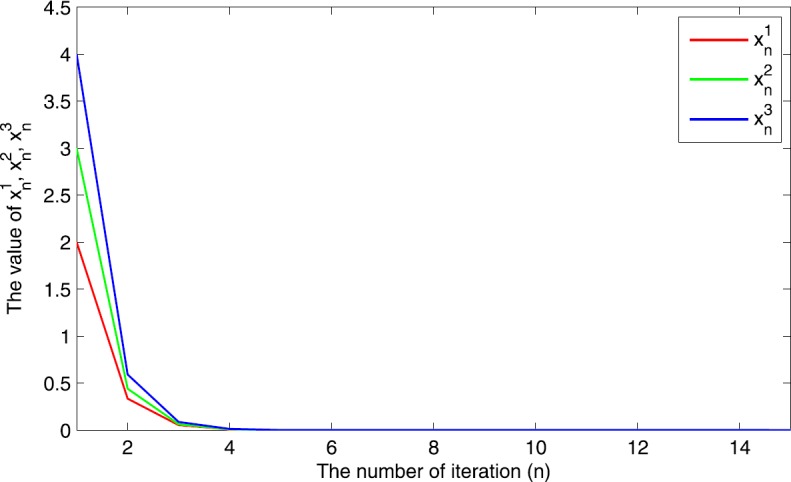
The convergence of $x_{n}$ with initial $x_{1}=(2,3,4)$

Figure 1 shows the convergence of the iterative sequence of Algorithm 4.2.

Solution: We can see from both Table 1 and Fig. 1 that the sequence $\{x_{n}\}$ converges to $(0, 0, 0)$, that is, $(0, 0, 0)$ is the solution in Example 4.1. In addition, it is also easy to check from Example 4.1 that $\bigcap^{N}_{i=1}F(T_{i})\cap\varGamma\cap \operatorname{VI}(C, D)=\{ (0, 0, 0)\}$. Therefore, the iterative algorithm of Theorem 3.1 is well-defined and efficient.

## Results and discussion

In this paper, we propose a new iterative scheme for finding a solution of SMVIP (1.3) with the constraints of a variational inequality and a fixed point problem of a finite family of strict pseudo-contractions in real Hilbert spaces. Moreover, we prove a strong convergence theorem for this iterative scheme.

In our main result, we not only give the definite domains and ranges of $B_{1}$ and $B_{2}$ to make sure our iterative scheme (1.7) well-defined, but also modify the iterative scheme (1.6) of Jitsupa et al. by adding the projection operator. Our result can be applied to finding a common solution of SMVIP (1.3), VIP (2.7), and fixed point problem of a finite family of strict pseudo-contraction mappings instead of SVIP (1.2) and fixed point problem of a finite family of strict pseudo-contraction mappings. Thus, our result improves and extends the result in [1].

## Conclusions

In this paper, we first propose a modified iterative scheme (1.7) and then prove the strong convergence of the sequence $\{x_{n}\}$ generated by (1.7) to a common solution of SMVIP (1.3), VIP (2.7), and a fixed point problem under suitable conditions. Finally, we give a numerical example to support our strong convergence result. As a result, our result includes, improves, and enriches the corresponding ones announced by some others, see, e.g., [1, 12, 13].

## Experimental

A numerical experiment is provided to support our iterative scheme in Algorithm 4.2, Table 1 and Fig. 1 above indicate the strong convergence of Algorithm 4.2. Therefore, our the iterative algorithm of Theorem 3.1 is well-defined and valid.
